# Clumped isotopes of methane trace bioenergetics in the environment

**DOI:** 10.1126/sciadv.adu1401

**Published:** 2025-06-25

**Authors:** Jiarui Liu, Edward D. Young, André Pellerin, Jeanine L. Ash, Gerard T. Barrett, Xiahong Feng, Peter R. Girguis, Sebastian J. E. Krause, William D. Leavitt, Kyla Murphy, Qianhui Qin, Andreas Teske, David L. Valentine, Katey Walter Anthony, Tina Treude

**Affiliations:** ^1^ Department of Earth, Planetary and Space Sciences, University of California, Los Angeles, CA 90095, USA.; ^2^ Institut des sciences de la mer, Université du Québec à Rimouski, Rimouski, Québec G5L 3A1, Canada.; ^3^ Department of Earth, Environmental and Planetary Sciences, Rice University, Houston, TX 77005, USA.; ^4^ CHRONO Centre for Climate, the Environment and Chronology, School of Natural and Built Environment, Queen’s University Belfast, Belfast BT9 6AX, UK.; ^5^ Department of Earth Sciences, Dartmouth College, Hanover, NH 03755, USA.; ^6^ Department of Organismic and Evolutionary Biology, Harvard University, Cambridge, MA 02138, USA.; ^7^ Earth Research Institute, University of California, Santa Barbara, CA 93106, USA.; ^8^ Department of Chemistry, Dartmouth College, Hanover, NH 03755, USA.; ^9^ Department of Earth Science, University of California, Santa Barbara, CA 93106, USA.; ^10^ Interdepartmental Graduate Program in Marine Science, University of California, Santa Barbara, CA 93106, USA.; ^11^ Department of Earth, Marine, and Environmental Sciences, University of North Carolina, Chapel Hill, NC 27599, USA.; ^12^ Marine Science Institute, University of California, Santa Barbara, CA 93106, USA.; ^13^ Water and Environmental Research Center, University of Alaska Fairbanks, Fairbanks, AK 99775, USA.

## Abstract

Methane is a major greenhouse gas and a key component of global biogeochemical cycles. Microbial methane often deviates from isotope and isotopolog equilibrium in surface environments but approaches equilibrium in deep subsurface sediments. The origin of this near-equilibrium isotopic signature in methane, whether directly produced by methanogens or achieved through anaerobic oxidation of methane (AOM), remains uncertain. Here, we show that, in the absence of AOM, microbial methane produced from deep-sea sediments exhibits isotopolog compositions approaching thermodynamic equilibrium due to energy limitation. In contrast, microbial methane from salt marsh and thermokarst lakes exhibits significant hydrogen and clumped isotopic disequilibrium due to high free-energy availability. We propose that clumped isotopologs of methane provide a proxy for characterizing the bioenergetics of environments for methane production. Together, these observations demonstrate methane clumped isotopes as a powerful tool to better understand the relation between methane metabolisms and the energy landscape in natural environments.

## Teaser

Clumped isotopes of methane provide a proxy for the bioenergetics of methanogenesis in natural environments.

## INTRODUCTION

Methane is a potent greenhouse gas emitted from both anthropogenic and natural sources (*1*, *2*). Microbial methanogenesis contributes more than half of annual emissions to the atmosphere (*3*, *4*). The strong isotopic discrimination imparted by methanogenesis has driven the widespread application of conventional stable carbon (^13^C/^12^C) and hydrogen (D/H) isotope ratios for distinguishing the origins of methane (*5*). Conventional stable isotope measurements of methane have become a cornerstone of global methane budgets and the assessment of its climatic impacts (*4*, *6*). Yet, a major limitation of this conventional isotope approach is the overlap in isotope signals from distinct sources of methane (*7*). The recent development and application of doubly substituted “clumped” isotopologs (i.e., ^13^CH_3_D and ^12^CH_2_D_2_) have emerged as a powerful tool for characterizing sources and sinks of methane (*8*–*12*). When the distribution of carbon and hydrogen isotopes among methane molecules is consistent with thermodynamic equilibrium, clumped isotopologs record the formation temperatures of thermogenic methane, for example, in natural gas reservoirs (*8*, *10*, *13*). In microbial ecosystems, methane clumped isotopologs often exhibit departures from equilibrium (i.e., disequilibrium) in surface environments but near-equilibrium signals in deep subsurface sediments (*9*, *10*, *14*–*19*). However, the near-equilibrium isotopolog signature imparted by microbial metabolisms is not well understood. In deep subsurface sediments, limited substrates, and thereby limitations of free energy availability during methanogenesis may lead to isotopolog compositions approaching equilibrium (*9*, *14*, *20*–*24*). Supporting this, a recent laboratory culture experiment revealed that both low H_2_ and high dissolved methane concentrations are key factors limiting the energy available for hydrogenotrophic methanogenesis in these environments (*24*). Yet, additional processes, such as anaerobic oxidation of methane (AOM), may also contribute to the expression of isotopic equilibrium in methane (*15*, *25*–*28*).

To test whether methane near thermodynamic equilibrium in natural environments (i) can be produced by methanogens, (ii) is influenced by energy landscape (where the availability and distribution of energy influence metabolic processes), and (iii) is produced in the absence of anaerobic oxidation, we examined methane sampled from diverse systems, including freshwater lakes, brackish wetlands, and marine basins (table S1 and fig. S1). The energy-rich systems investigated in this study include surface sediments from the Carpinteria salt marsh in California and thermokarst lakes in Alaska. By contrast, the energy-limited systems comprise subsurface sediments from the Santa Barbara Basin, as well as both surface and subsurface sediments from the Guaymas Basin, with overlying water depths of 580 and ~2000 m, respectively (see the Supplementary Materials).

We collected on-site methane gas in the field, where methanogenesis rates were shown to significantly exceed AOM (*P* < 0.01). The on-site methane samples from the two marine basins were collected below the sulfate-methane transition zone (SMTZ). In the Guaymas Basin, sediment ages range from about 158 to 295 thousand years (ka) at depths of 109 to 204 m below sea floor, while in the Santa Barbara Basin, sediment ages range from about 1.5 to 1.8 ka at depths of 1.7 to 2.1 m. The latter is comparable to studied near-shore sites where cell-specific metabolic rates in sediments of ~1 ka have been shown to approach those of the deep biosphere (*29*). The rate constant of organic matter degradation decreases following a power-law relationship with age, meaning that older organic matter in deep-sea sediments is far less reactive than fresh material from surface aquatic environments (*30*, *31*). Thus, the organic matter in these sediments is highly degraded and refractory, indicating energy-limiting conditions (*29*, *30*). To connect methanogenesis in natural environments with laboratory observations, we performed incubation experiments to study methanogenesis in these sediments while suppressing potential AOM (see Materials and Methods). As a precaution, we amended these samples with AOM inhibitors in a manner that did not alter natural substrate concentrations. We quantified the abundance of the two most common methane clumped isotopologs Δ^13^CH_3_D and Δ^12^CH_2_D_2_ relative to a random isotopic distribution (*10*). This approach enabled us to link methane clumped isotope signatures to formation processes (*32*).

## RESULTS

### Departures from equilibrium reflecting kinetics

**Fig. 1.**
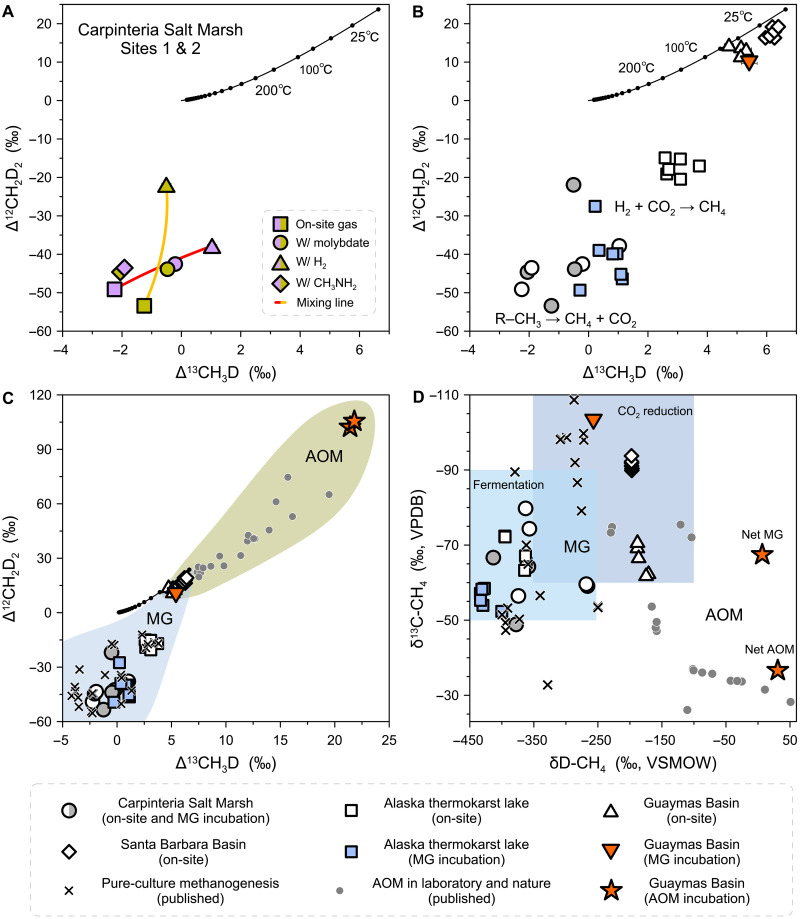
Isotopolog compositions of methane samples. (**A** and **B**) Δ^13^CH_3_D plotted against Δ^12^CH_2_D_2_ for methanogenesis samples from the Carpinteria salt marsh (A) and all study sites (B). In (A), light purple and dark yellow symbols depict samples from sites 1 and 2, respectively. The red and orange curves represent mixing lines between the two methanogenic end-members, the methylotrophic and hydrogenotrophic pathways. The solid black curves depict theoretical thermodynamic equilibrium abundances of methane isotopologs, along with corresponding temperatures. (**C**) Illustration outlining clumped isotope signatures of methanogenesis (MG) and AOM. The orange triangle represents the slurry incubation from the Guaymas Basin conducted with AOM inhibitors, permitting only MG. The orange stars represent slurry incubations from the same site conducted without AOM inhibitors, allowing both MG and AOM to occur simultaneously, with the lower-left star indicating net AOM and the upper-right star indicating net MG. The cross symbols represent published MG data from pure culture incubations, while the gray dots depict published kinetically driven AOM data from slurry incubations and natural samples (*10*, *17*, *54*, *55*). The light blue and dark yellow fields encompass all the MG and AOM data, respectively. (**D**) δ^13^C plotted against δD. The genetic fields for methane sources follow (*5*, *7*). The legend for (B) to (D) is displayed below the plots. Error bars of 1 SE are mostly encompassed by individual data points.

Methanogens from laboratory cultures typically produce methane with pronounced kinetic isotope and isotopolog signals, except under conditions of low substrate availability and high dissolved methane concentrations (*9*, *10*, *17*, *24*, *25*, *33*). Similarly, methane from the Carpinteria salt marsh (California) and thermokarst lakes (Alaska) yielded disequilibrium isotopolog compositions with low Δ^13^CH_3_D and Δ^12^CH_2_D_2_ values, down to −2.3 and −53.4 per mil (‰), respectively (Fig. 1, A and B). Control incubations with a tracer eliminated the possibility of concurrent AOM (figs. S2 and S3 and table S2). Therefore, these values reflect kinetic isotope effects and associated combinatorial effects during methanogenesis under energy-rich conditions (*10*, *17*, *33*–*37*).

Using the Carpinteria salt marsh as an example, we investigated the isotopolog effects of different methanogenic pathways (Fig. 1A and fig. S4). First, methane collected from sediments (hereafter referred to as on-site methane) primarily originates from methylotrophic methanogenesis within sulfate-rich sediments (see Supplementary Text) (*38*, *39*). Methylotrophic methanogenesis is further confirmed by minor differences in methane isotopolog compositions observed between methylamine (CH_3_NH_2_) incubations and on-site methane (Fig. 1A). These differences likely arise from distinct clumping signals of the precursor methyl groups in natural and synthetic methyl compounds used in situ and in laboratory incubations, respectively (*40*). Methylotrophic methanogenesis yields substantial free energy, with the molar Gibbs free energy of the reaction (Δ$\hat{G}$_rxn_, hereafter termed Δ*G*) in the range of ~−60 kJ mol^−1^ per carbon transferred (table S3). The extremely low Δ^12^CH_2_D_2_ values, ~−45‰, result from substantially different δ*D* between water and methyl groups, which serve as hydrogen sources contributing to the formation of methane molecules. This process is termed the exogenous combinatorial effect, driven by distinct hydrogen sources both inside and outside the cell (*34*). In Carpinteria salt marsh sediments where we introduced excess H_2_, we observed a greater fraction of methane from hydrogenotrophic methanogenesis. This pathway results in less negative Δ^12^CH_2_D_2_ values because the four hydrogen atoms that form methane are derived from water during hydrogenotrophic methanogenesis (*33*). In this context, the endogenous combinatorial effect is constrained to intracellular reactions occurring entirely within the cell, where the hydrogen isotopic compositions of intermediate metabolites differ due to isotope fractionations during methanogenesis (*17*, *35*–*37*). Last, we used molybdate and aqueous sulfide to inhibit the reduction of sulfate and iron(III) associated with AOM, respectively, allowing in situ H_2_ to be consumed by methanogens instead of sulfate and iron reducers. The outcome is the simultaneous occurrence of methylotrophic and hydrogenotrophic methanogenesis in sediment incubations, as evidenced by their methane isotopolog compositions falling along a mixing trajectory between the two methanogenic pathways (Fig. 1A). Therefore, methane clumped isotopes provide a useful tool for tracing the pathways of methane production in natural environments.

The evolution of methanogenic pathways and the resulting shifts in methane isotopolog compositions are particularly evident in Alaska thermokarst lakes. Data from the studied lakes revealed two distinct groups: in vitro incubations of surface sediment exhibiting more negative Δ^13^CH_3_D and Δ^12^CH_2_D_2_ values and on-site deep subsurface gas with less negative values (Fig. 1B). Methane production in low-sulfate lake sediments is typically dominated by acetoclastic methanogenesis, especially at low in situ temperatures (*41*, *42*). Laboratory incubations of near-surface sediment from the study site highly favored this pathway (fig. S3C) (*43*), producing strongly negative Δ^12^CH_2_D_2_ values reflecting combinatorial effects from hydrogen sources in acetate and water. Deeper in the sediment, where the buried organic matter becomes less degradable and the microbial communities face increased energy limitations, hydrogenotrophic methanogenesis becomes more dominant (fig. S3C) (*44*, *45*). This shift is evident in the less negative Δ^12^CH_2_D_2_ values observed in deeper incubation and on-site subsurface methane (Fig. 1B and fig. S5). Diminished availability of labile organic matter and the resultant reduction in free energy availability downcore may have fostered reaction reversibility (i.e., the ratio of the reverse to forward rates of reaction) in methanogenesis, thereby also contributing to less negative isotopolog compositions (*14*).

Time-dependent changes in Δ^13^CH_3_D versus Δ^12^CH_2_D_2_ space in the same sediment incubations from Alaska thermokarst lakes provide further validation of the role of organic matter. Surface sediments were incubated for 2 years (2020–2022). After this initial incubation, the headspace was sampled and then flushed with argon. The same sediments were then reincubated under identical conditions for an additional 2 years (2022–2024). Methane generated in the second incubation exhibited Δ^12^CH_2_D_2_ values less negative than those in the first incubation, indicating a trajectory toward equilibrium (fig. S5). We propose that this shift reflects the aging of organic matter, resulting in diminished availability of the labile fraction over time. The 4-year timescale is comparable in magnitude to the age of this thermokarst lake (50–70 years) (*46*). Before the lake formed between 1949 and 1967, the sediment existed as permafrost, retarding the degradation of organic matter under frozen conditions until the sediment eventually thawed (*43*). Together, we suggest that methanogenesis evolution in Δ^13^CH_3_D versus Δ^12^CH_2_D_2_ space is governed by the changing organic matter dynamics with depth within lake sediments, reflecting a convolution of pathway transition from acetoclastic to hydrogenotrophic methanogenesis and possibly an increase in reaction reversibility attributable to limitations of free energy. Overall, in energy-rich surface environments, methane isotopolog compositions are primarily controlled by kinetic and combinatorial isotope effects (*32*).

### Methane approaching thermodynamic equilibrium under energy-limited conditions

Deep-sea marine sediments, where methane is predominantly produced through hydrogenotrophic methanogenesis under energy-limited conditions (*47*, *48*), provide further support for our proposition. For instance, methane collected below the SMTZ in the Santa Barbara Basin exhibits apparent formation temperatures of 7° to 19°C and 27° to 49°C based on Δ^13^CH_3_D and Δ^12^CH_2_D_2_ values, respectively (Fig. 1B). These apparent formation temperatures approach the in situ temperature of 6°C. Porewater methane modeling indicates the presence of active methanogenesis (fig. S6). Sulfate reduction, if occurring, proceeds at an exceptionally slow rate below the SMTZ (*49*). The elevated concentrations of aqueous sulfide, coupled with the absence of dissolved Fe^2+^, preclude the possibility of microbial iron(III) reduction of highly reactive iron oxides, although microbial iron reduction may still occur at an exceptionally slow rate with poorly reactive iron(III) minerals (fig. S6) (*50*). With negligible electron-accepting processes to accommodate AOM, we conclude that the near-equilibrium methane isotopolog compositions of the Santa Barbara samples result from a slow rate of methanogenesis (0.2 nmol cm^−3^ day^−1^) under energy-limited conditions (Δ*G* ranging from −22.6 to −14.7 kJ mol^−1^ C), characterized by a low hydrogen availability (4.7 ± 0.7 nM; fig. S6 and table S3).

The samples collected from the Guaymas Basin offer a unique perspective for comparing surface and deep subsurface methanogenesis. Both environments are characterized by high dissolved methane concentrations, with active methane bubbling from the surface sediment in situ and substantial postrecovery methane degassing from deep subsurface sediments. To completely rule out AOM, we conducted slurry incubations of surface sediment (0 to 20 cm) from the Guaymas Basin with molybdate and aqueous sulfide at 20°C. Before incubation, natural methane in the samples was removed by flushing with argon three separate times. The incubation lasted for 2 years. Notably, the AOM rate, as determined by ^14^C radiotracer, was below the detection limit (table S2). The methane produced falls near the equilibrium curve, with apparent formation temperatures of 37° ± 10°C and 122° ± 14°C indicated by Δ^13^CH_3_D and Δ^12^CH_2_D_2_ values, respectively (Fig. 1B), although minor disequilibrium remains. To our knowledge, this result marks the first clear demonstration that methanogens from natural environments can produce methane approaching isotopolog equilibrium in Δ^13^CH_3_D versus Δ^12^CH_2_D_2_ space independently of AOM.

In addition, on-site methane collected below the SMTZ from a drilling core in the Guaymas Basin exhibits clumped isotopolog compositions near equilibrium at relatively low temperatures, with apparent Δ^13^CH_3_D and Δ^12^CH_2_D_2_ formation temperatures being 40° to 62°C and 64° to 95°C, respectively, compared with measured in situ temperatures of 18° to 31°C (Fig. 1B). While the potential role of AOM in this drilling core cannot be entirely discounted, AOM rates were either near or below the detection limit in analogous deep biosphere sediments (*51*). The close agreement between the AOM-free incubation and the on-site samples suggests that AOM, if present, does not influence Δ^13^CH_3_D and Δ^12^CH_2_D_2_ values in this setting (Fig. 1B). Overall, in energy-limited subsurface environments, methane isotopolog compositions are primarily governed by equilibrium isotope effects (*32*).

**Fig. 2.**
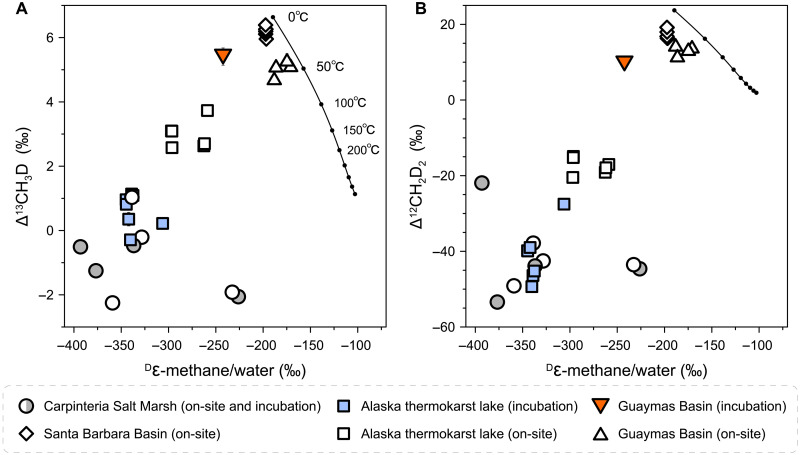
The degree of hydrogen and clumped isotopic disequilibrium in methane. Hydrogen isotope fractionation between methane and associated water is plotted against Δ^13^CH_3_D (**A**) and Δ^12^CH_2_D_2_ (**B**). The solid black curves depict thermodynamic isotopic equilibrium, with the ^D^ε-methane/water calibration given in (*20*). Statistical correlation analysis yields *P* values less than 0.01 for the data presented in both panels. Error bars of 1 SE are mostly encompassed by individual data points.

By combining datasets from all the investigated systems, we applied metabolic-isotopic and isotopolog flow network models of hydrogenotrophic methanogenesis to our data (*35*, *36*, *52*). We find that our hydrogenotrophic data are largely consistent with these model predictions (fig. S7). We extend our analysis to compare the degree of D/H fractionation between methane and environmental water (^D^ε-methane/water) against the isotopolog data. The significant positive correlation between ^D^ε-methane/water and methane isotopolog compositions underscores a strong connection between hydrogen isotope and clumped isotopolog disequilibrium (Fig. 2, A and B) (*9*). On the other hand, microbial methane in deep-sea environments tends to approach hydrogen isotopic equilibrium with associated waters at or near the temperatures indicated by the Δ^13^CH_3_D and Δ^12^CH_2_D_2_ values, suggesting the occurrence of both intra- and interspecies isotopic equilibrium (Fig. 2, A and B).

### The isotopolog effects of concurrent methanogenesis and AOM

In the SMTZ of marine sediments, methane is oxidized by anaerobic methanotrophic archaea (*53*). To explore the isotopolog effects of concurrent methanogenesis and AOM, we conducted two parallel incubations using separate aliquots of sediment slurries from the same methane-seeping surface sediment in the Guaymas Basin. One incubation was carried out under an argon headspace and the other under a methane headspace, both without the addition of AOM inhibitors. On the basis of methane concentrations and ^14^C radiotracer analysis, net methanogenesis with simultaneous AOM was identified in the slurry with an argon headspace (table S2 and fig. S8A). The produced methane displays remarkably high Δ^13^CH_3_D and Δ^12^CH_2_D_2_ values, reaching up to 21.8 and 105.4‰, respectively—representing, we believe, the highest methane isotopolog compositions reported to date (Fig. 1C). In the slurry containing a methane headspace, net AOM with simultaneous methanogenesis resulted in nearly identical, elevated Δ^13^CH_3_D and Δ^12^CH_2_D_2_ values, compared to the argon headspace incubation (Fig. 1C). Similar trajectories in Δ^13^CH_3_D versus Δ^12^CH_2_D_2_ space have been observed in both laboratory incubations and natural samples associated with kinetically driven AOM (*54*, *55*).

As discussed in the previous section, the same surface sediment from the Guaymas Basin, treated with AOM inhibitors, produced methane approaching isotopolog equilibrium (Fig. 1B). The highly positive signals observed in the incubations without AOM inhibitors suggest that the incubation conditions may allow AOM to express stronger kinetic isotopolog effects, compared to methanogenesis. In these incubations with active methane cycling, methanogenesis served solely as a source of methane, generating methane in isotopolog equilibrium, while changes in isotopolog composition—and the resulting highly positive signals—were driven by AOM. A closed-system time-evolution isotope model with concurrent methanogenesis and AOM reproduced these exceptionally high values by incorporating the kinetic clumped isotopolog fractionation factors reported in our previous AOM-only incubations (fig. S8) (*55*). The closed-system model suggests that concurrent methanogenesis and AOM in both slurries have reached a steady state. Even in the slurry with an argon headspace where the methanogenesis rate exceeded the AOM rate, the expressed isotopolog effects remained dominated by AOM-driven fractionation, consistent with our model predictions (fig. S8). Put differently, in a closed system with concurrent methane production and oxidation, the steady-state isotopolog compositions are strongly influenced by the fractionation associated with the oxidation reaction, even when the production rate substantially exceeds the oxidation rate.

Conversely, recent studies have shown that AOM drives methane isotopologs toward equilibrium under conditions of low thermodynamic driving force (*15*, *55*). Therefore, the presence of near-equilibrium methane in subsurface environments has been interpreted as either a consequence of methanogenesis with high reversibility or the involvement of AOM (*9*, *14*, *15*, *22*, *25*). Given that the initial step of AOM and the final step of methanogenesis are catalyzed by the same enzyme, methyl-coenzyme M reductase (Mcr), we suggest that the near-equilibrium methane isotopolog signatures result from the Mcr-catalyzed intracellular isotope exchange operating under conditions of near-threshold free energy, allowing for either net methanogenesis or AOM (*24*, *36*, *55*, *56*). In this case, there will always—inevitably—be sufficient back flux of either dominant methane-cycling reaction to drive isotopologs toward equilibrium. Therefore, methane exhibiting intra- and interspecies isotopic equilibrium essentially indicates the dominance of Mcr-catalyzed isotope exchange, near equilibrium, regardless of whether the net process is methanogenesis or AOM. When the thermodynamic driving force is elevated, methanogenesis and AOM can generate more negative and positive isotopolog signatures, respectively, in comparison to isotopolog equilibrium (Fig. 1C). By synthesizing isotope data from previous studies, we illustrate that methane clumped isotopologs offer deeper insight into anaerobic microbial metabolisms compared to conventional isotope measurements (Fig. 1, C and D) (*32*).

## DISCUSSION

Microbial methane in surface environments typically displays deviations from isotopolog equilibrium, while it tends to approach equilibrium in deep subsurface sediments. The origin of this near-equilibrium methane, whether directly produced by methanogens or achieved through AOM, has long been a subject of debate. Building on the results from earlier sections, we show that methane near thermodynamic equilibrium in natural environments can be produced solely by methanogens, independent of AOM, and is shaped by the energy landscape.

**Fig. 3.**
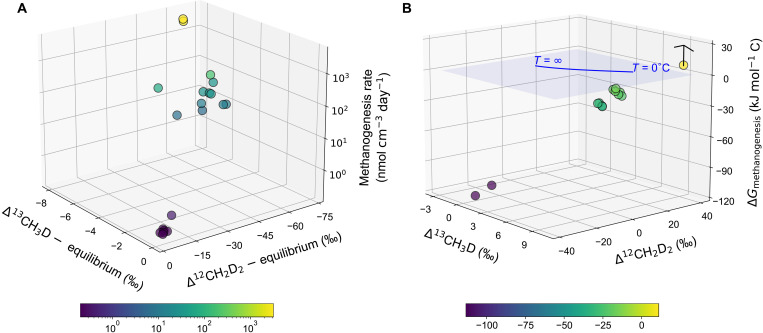
Methanogenesis rate and Gibbs free energy plotted against isotopolog data. The departures from isotopolog equilibrium in (**A**) are defined as the difference between measured isotopolog compositions and equilibrium isotopolog compositions at in situ or incubation temperatures. In (**B**), only the Δ*G* of hydrogenotrophic methanogenesis are included for comparison, encompassing on-site samples from the Santa Barbara and Guaymas Basins, as well as Carpinteria salt marsh incubations with an H_2_ headspace. The published AOM isotopolog data are averaged (*54*, *55*) and presented with a published Δ*G* value of 11 kJ mol^−1^ C with respect to hydrogenotrophic methanogenesis (*60*), offering a conservative estimate. Note that a positive Δ*G* is applied to AOM to allow comparison with isotopolog data from hydrogenotrophic methanogenesis, albeit with some caveats. The solid blue curve depicts theoretical thermodynamic equilibrium abundances of methane isotopologs, along with corresponding temperatures. The blue plane represents the surface at which Δ*G* equals zero. The symbols are color-coded to reflect variations along the *z* axis, with color bars shown below each plot. Statistical correlation analysis yields *P* values less than 0.01 for the data presented in both panels. This figure is also shown in fig. S9 with more details.

We broaden our analysis to evaluate the fundamental mechanisms that dictate the degree of isotopolog disequilibrium. Notably, a significant positive correlation is observed between methanogenesis rate and departures from isotopolog equilibrium (Fig. 3A and fig. S9). This finding aligns with the notion that a high rate of methanogenesis exhibits pronounced kinetic and combinatorial imprints, whereas a low rate of methanogenesis results in near-equilibrium signatures in methane (*14*). The difference in departures from isotopolog equilibrium caused by high and low rates of methanogenesis is inherently driven by the reactivity of organic matter in the environment. Ramped pyrolysis analysis further supports our proposition. Thermograms and activation energy distributions of organic carbon indicate that the Santa Barbara Basin sediments have a somewhat higher proportion of refractory organic carbon compared to sediments from the salt marsh and thermokarst lake (fig. S10), leading to reduced substrate reactivity (*30*). As a result, the low availability and high recalcitrance of metabolizable substrates in deep-sea sediments maintain low rates of methanogenesis (*29*, *57*).

For the same pathway, all else being equal, a lower substrate concentration typically results in a more positive Δ*G*. Departures from isotopolog equilibrium can be related to the bioenergetics of a site using the principles of disequilibrium thermodynamics, in which rates of reaction are proportional to reaction affinity (*58*). Reaction affinity can be related to the forward and reverse reaction rates, *r*_f_ and *r*_rev_, respectively, using *A* = RTln(*r*_f_/*r*_rev_). Reaction affinity is defined as *A* = −∑ν*_i_* μ*_i_*, where ν*_i_* is stoichiometric coefficient for species *i* in a reaction and μ*_i_* is the chemical potential. From the definition of chemical potential, we have *A* = RTln(*K*_Eq_/*Q*) = −Δ*G*, where *Q* is the measured activity quotient for the reaction and *K*_Eq_ is the equilibrium constant, and thus, Δ*G* = −RTln(*r*_f_/*r*_rev_). The measured activity quotients, and thus free energies, are indicators of the reversibility and rates of the reaction. In the case of hydrogenotrophic methanogenesis without syntrophic partners, a more positive Δ*G* indicates higher reversibility and a lower net methanogenesis rate. Note that the derivation relies on several assumptions that may not apply to certain nonelementary reactions, so deviations are expected in such cases (*59*).

A recent study cocultured a hydrogenotrophic methanogen with an H_2_-producing bacterium at 55°C in the laboratory (*24*). Under low hydrogen concentrations and high hydrostatic pressure, the produced methane achieved equilibrium for both carbon and hydrogen isotopes (*24*). While high pressure and the resulting elevated dissolved methane concentrations may facilitate the attainment of equilibrium, it is ultimately the change in Gibbs free energy that governs isotoplog equilibrium (*24*, *35*, *36*). Notably, the slurry incubation of Guaymas Basin sediment reached isotopic equilibrium at one atmospheric pressure (Fig. 1B), suggesting that high dissolved methane concentrations under elevated hydrostatic pressure are not a prerequisite for achieving isotopic equilibrium during methanogenesis.

**Fig. 4.**
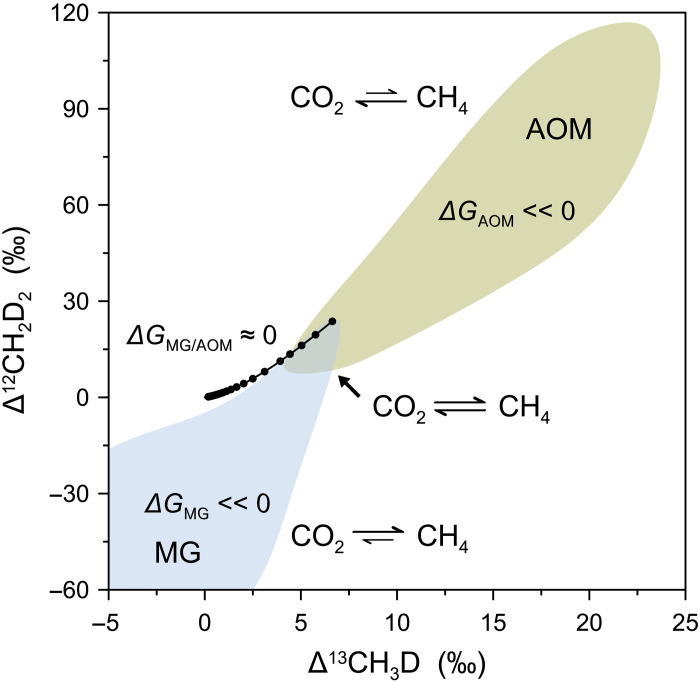
A schematic representation illustrating the connection between methane clumped isotope signatures and the free energy availability in anaerobic microbial metabolisms. The shaded regions are adapted from Fig. 1C. The solid black curve depicts theoretical thermodynamic equilibrium abundances of methane isotopologs. Reaction reversibility of MG and AOM is demonstrated using the CO_2_─CH_4_ system as an example.

To quantitatively address the energy hypothesis, we include the available Δ*G* values of hydrogenotrophic methanogenesis for comparison (Fig. 3B and figs. S9 and S11). In addition, we include published isotopolog data on AOM and assume that AOM is analogous to reversed hydrogenotrophic methanogenesis, expressing the Δ*G* of the environment in terms of the energy available for hydrogenotrophic methanogenesis (Fig. 3B) (*60*). Taking the data in combination, we observe a significant positive correlation between methane isotopolog compositions and Δ*G* values, with near-equilibrium isotopolog compositions aligning with Δ*G* values close to zero (blue plane in Fig. 3B). Therefore, we propose that the relative abundances of clumped isotopologs of methane provide a proxy for the bioenergetics of a site with respect to methanogenesis (Fig. 4). Although only hydrogenotrophic methanogenesis is presented here for comparison, we argue that isotopolog data are methanogenic pathway–independent proxies of the energetic state of methanogenic cells in the environment.

Furthermore, to directly calculate the Δ*G* of hydrogenotrophic methanogenesis, it is necessary to measure porewater hydrogen concentrations. The two most commonly used techniques, the headspace equilibration method and the extraction method, may produce systematic discrepancies due to technique-related challenges (see Supplementary Text) (*61*). Another complication arises from direct interspecies electron transfer. It was shown that methanogenic CO_2_ reduction in Swiss lake sediments is primarily driven by interspecies electron transfer in association with syntrophic bacteria, without the involvement of H_2_ as an electron shuttle, further complicating the calculation of Δ*G* (*62*). Consequently, this work offers a useful tool for characterizing the bioenergetics of environments for methane production, where estimates of substrate concentrations are not readily available to calculate Δ*G*. We conclude that clumped isotopes of methane provide deeper insight into the relation between methane metabolisms and the energy landscape in natural environments (Fig. 4).

Notably, this finding has broad implications for the study of methane biogeochemistry, the assessment of atmospheric methane budgets, and potential astrobiological applications. On a global scale, terrestrial microbial sources exhibit markedly negative Δ^12^CH_2_D_2_ values due to kinetic and combinatorial effects, while marine microbial sources have Δ^12^CH_2_D_2_ values near equilibrium (*9*, *14*, *15*, *22*, *34*). Here, we show that this difference is a direct consequence of the availability of chemical energy in these respective environments. Because of the very different Δ^12^CH_2_D_2_ values of these sources, recent measurements of Δ^12^CH_2_D_2_ values in atmospheric methane demonstrate that the primary microbial sources of methane in air must indeed be terrestrial and not marine, alongside contributions from fossil fuel emissions (*11*, *12*). Methane isotopologs thereby provide an additional constraint on global methane budgets.

The significance of clumped isotopologs in astrobiological applications lies in the fact that their distribution offers an intramolecular or intraphase isotope signal, without needing to reference other species (*63*). In contrast, stable isotope ratios typically require comparison with ratios in a different phase or molecule for interpretation. In other words, isotopic bond ordering, or “clumping,” is a tracer for the processes involved in the formation of molecules, as well as subsequent consumption or reequilibration (*17*, *32*). This unique characteristic makes clumped isotopologs a powerful tool for investigating methane formation and oxidation processes, both on Earth and other solar system bodies.

## MATERIALS AND METHODS

Four study sites were chosen to investigate the clumped isotopolog effects of natural methanogenesis, including the Carpinteria Salt Marsh Reserve, Alaska thermokarst lakes, the Santa Barbara Basin, and the Guaymas Basin (fig. S1 and table S1). Site description and sampling protocols of gas and sediment are provided in the Supplementary Materials. Rates of methanogenesis and AOM were determined under ex situ and in vitro conditions. The goal was to sample on-site methane gas where methanogenesis rates significantly exceeded AOM rates (*P* < 0.01) and to incubate sediment for methanogenesis with the addition of AOM inhibitors (*32*).

### Sediment slurry incubation

While methane gases collected in the field were chosen from natural settings characterized by low rates of AOM, the AOM rates were still on the order of tens to hundreds of picomoles per cubic centimeter per day in the salt marsh and thermokarst lake (figs. S2 and S3). Simultaneous AOM activity, even if low, complicates the interpretation of isotopolog data when studying the signatures of methanogenesis. Accordingly, we conducted sediment slurry incubation experiments to collect methane produced by indigenous methanogens, amended with AOM inhibitors but without alteration of the natural substrate concentration. To date, there is no inhibitor that specifically targets AOM without inhibiting methanogenesis simultaneously (e.g., 2-bromoethanesulfonate) (*64*). However, AOM is coupled to the reduction of electron acceptors, including but not limited to sulfate, nitrate, and iron oxides (*53*, *65*). In anoxic sediments, nitrate is the first electron acceptor to be depleted, driven by the high energy yield of denitrification, occurring before the accumulation of methane begins (*66*). We anticipate the absence of nitrate in our sediment slurries. Sulfate reduction is readily inhibited by the addition of molybdate (*64*). Iron reduction can be prevented by the addition of sulfide because aqueous sulfide reacts with iron oxides, resulting in the absence of microbially reducible Fe(III) in the system (*50*, *67*, *68*). However, sulfide toxicity at sulfide levels greater than 3 mM may substantially inhibit methanogenic activity (*69*). Therefore, the slurries were initiated with sulfide concentrations at or below 3 mM, and we ensured the presence of aqueous sulfide at the end of the incubation period.

For surface sediments from the salt marsh and the Guaymas Basin, artificial seawater medium was prepared without sulfate following the method in (*70*). Sediment slurries were made by mixing sediment with seawater medium at a 1:1 to 1:2 (v/v) ratio in 500-ml borosilicate bottles, leaving a 20- to 60-ml headspace. Concurrently, molybdate was introduced to reach a final concentration of 20 mM. The headspace was flushed with pure argon gas for three separate 5-min intervals and vigorously shaken between the intervals to remove all traces of methane and oxygen. The bottles were kept in the dark and shaken on a weekly basis. The incubation temperature was 20°C for the salt marsh and Guaymas Basin slurries, which closely approximated the in situ condition. These slurries were used to study methanogenesis under the simultaneous inhibition of iron and sulfate reduction and without amendment of methanogenic substrates. Furthermore, we set up three additional sets of slurries for salt marsh sediments following the procedure above but with the addition of methanogenic substrates to study the isotopolog signatures of common methanogenic pathways. These substrates included 150-kPa hydrogen gas in the headspace, 40 mM acetate, and 100 mM monomethylamine (referred to as methylamine hereafter), respectively, reflecting standard methanogen pure culture incubations (*33*). Slurry volumes for these incubations were smaller (40 to 200 ml) as methane production was faster compared to slurries without substrate addition.

Surface sediment from the Guaymas Basin, collected from an active methane seep, provided an ideal system for investigating the isotopolog effects of concurrent methanogenesis and AOM, due to the high biomass of methanogenic and anaerobic methanotrophic archaea (*71*). Following the same procedure as above but without molybdate and sulfide addition, we established another two sets of Guaymas Basin slurries. We aimed to investigate the isotopolog effects of net methanogenesis and net AOM when methanogenesis and AOM were occurring simultaneously. One set was flushed with argon to remove methane in the slurry, whereas the headspace of the other set was replenished with ~200 kPa of tank methane (Airgas).

Alaska lake sediments were flushed with argon after fieldwork and incubated at 4°C without the addition of liquid medium or inhibitors, as porewater sulfate concentrations (<30 μM) were near or below the detection limit (*43*). Moreover, Lotem and coauthors (*72*) found that the reduction of iron(III) was not coupled to AOM and demonstrated that the AOM signals in the incubation experiments may result from enzymatic reversibility (“back-flux”) during methane production rather than thermodynamically favorable AOM. Specifically, sediments from units 2 and 4 were incubated for 2 years (2020–2022). After this initial incubation, the headspace was sampled and then flushed with argon. The same sediments were then reincubated under identical conditions for an additional 2 years (2022–2024). Sediment from unit 1, collected from a deeper layer, was incubated continuously for 4 years (2020–2024).

### Geochemical analyses

Porewater was separated from sediment by centrifugation in argon-flushed centrifuge tubes. The supernatant from sediment slurries was sampled using a long needle through the rubber stopper without opening the bottle. The concentrations of dissolved solutes in sediment porewater (ex situ) and sediment slurry incubations (in vitro) were immediately measured after sampling, with the exception of sulfate, which was preserved at −20°C. For dissolved sulfide and iron analyses, porewater was immediately fixed with 5% zinc acetate solution (1:1, v/v) and 1% ascorbic acid solution (100:1, v/v), respectively. Dissolved sulfide and ferrous iron concentrations were determined by the methylene blue method (*73*) and the ferrozine assay (*74*), respectively, using a spectrophotometer (Shimadzu, UV-1800). The detection limits for sulfide and ferrous iron were 1 μM, exhibiting relative standard deviation (RSD) of less than 5%. Alkalinity was determined by acid titration with a Metrohm 876 Dosimat Plus. Sulfate concentrations were analyzed by ion chromatography (Metrohm 761). Alkalinity and sulfate concentrations were calibrated against standard seawater from the International Association for the Physical Sciences of the Oceans (IAPSO) with RSD better than 2%. The detection limit for sulfate was about 30 μM with dilution. Furthermore, pH was determined by a pH meter (VWR, sympHony B10P).

Alkane concentrations (C_1_ to C_4_) in gas samples were determined using a Shimadzu gas chromatograph (GC; GC-2014) with a packed HayeSep-D column and a flame ionization detector. Helium was used as the carrier gas at a flow rate of 35 ml/min. The column temperature was set at 120°C and held for 6 min and then increased up to 160°C at 20°C/min and held for 4 min. Alternatively, when only C_1_ was analyzed, the column temperature was set at 80°C with a helium flow of 15 ml/min. C_1_ to C_4_ alkane concentrations were calibrated against calibration standards (Gasco Precision Calibration Gas). The RSD was better than 5%, and the detection limit was about 1 parts per million (ppm).

Porewater hydrogen concentrations were determined in triplicate onboard for gravity core sediment from the Santa Barbara Basin, following a headspace equilibration technique (*75*). Sediment samples (6 cm^3^) were enclosed in 12-ml serum vials with an O_2_-free nitrogen headspace. The samples underwent a 54-hour incubation in the dark at 6°C for equilibration. Following this incubation period, the H_2_ concentration in the headspace was determined using Peak Performer 1 gas chromatography with a reducing compound photometer. Measurements on replicate standards typically exhibited a precision of less than 5%. The partial pressure values of the gas phase obtained through chromatographic analysis were converted to porewater concentrations using solubility constants corrected for temperature and salinity (*76*).

The hydrogen isotopic composition of water from slurry incubations and sediment porewater was analyzed following the method described in (*77*). Water hydrogen isotopic ratios (δ*D*) were measured using an H-Device, in which water was reduced by hot chromium (850°C), and the resulting hydrogen gas was measured by a Thermo Delta Plus XL isotope ratio mass spectrometer (IRMS). Isotopic ratios (D/H) are reported in δ-notation relative to the Vienna standard mean ocean water (VSMOW) standard. Analytical precision for δ*D* is <0.5‰ (1σ) based on replicate analyses of laboratory standards.

To determine the thermochemical stability of organic matter, we performed ramped pyrolysis/oxidation analysis on sediment samples (*78*). The system is made up of a carrier gas supply unit, pyrolysis furnaces, and an infrared CO_2_ analyzer. Defrosted sediment was acidified overnight using 0.5 N HCl to remove carbonates, washed three times using Milli-Q water, and dried at 40°C before analysis. Acid-rinsed sediment containing 0.8 to 1.1 mg of organic carbon was loaded into the inner quartz reactor and operated in suboxidation mode. In this mode, the gas supply to the inner tube consisted of helium (27 ml min^−1^) and diluted oxygen (3 ml min^−1^; 5% oxygen/95% nitrogen). An additional oxygen (5 ml min^−1^) was introduced directly to the outer quartz tube, oxidizing pyrolytic products from the inner quartz reactor to CO_2_ downstream. Samples underwent pyrolysis in the upper furnace, with temperatures ramping from 70° to 1050°C at a constant ramping rate of 5°C min^−1^, while the lower furnace was maintained at 800°C. The carrier gas with evolved CO_2_ then passed through the CO_2_ analyzer (Sable, CA-10), and instantaneous CO_2_ concentrations were recorded. CO_2_ concentrations were plotted against temperatures to generate thermograms, displaying the temperature-dependent decomposition of organic carbon. The precision of the oven ramping rate and CO_2_ concentration measurements were better than 1% and 5 ppm, respectively.

To quantitatively compare organic carbon bond strengths between samples, observed thermograms were converted to activation energy (*E*) distributions using a Python package (*79*). A regularized inverse method was used to estimate the distribution of organic carbon activation energy using serial oxidation. *E* distributions were calculated by finding the inverse solution to a set of parallel, non-isothermal, first-order kinetic decay reactions. *E* reflects the energy required to fully oxidize each carbon atom when exposed to a particular oxidation reaction pathway and is a suitable quantitative proxy for bond strength (*79*). The mean value of *E* (μ_E_) and the fraction of organic carbon within *E* values higher than 190 kJ mol^−1^ (*f*_E > 190_) were calculated accordingly. Higher μ_E_ and *f*_E > 190_ values mean the sediment contains more refractory or less labile organic carbon (*78*).

### Rate measurements and modeling of methanogenesis and AOM

Methane concentrations in the headspace of slurry incubations were monitored by gas chromatography throughout the incubation period. Using the known volume and porosity of the slurry, the amounts of dissolved methane were calculated using Henry’s law and the Bunsen solubility coefficient (*80*). Methanogenesis rates were then determined by combining the total amounts of methane in the headspace and the liquid phase and their development over time.

After extracting an aliquot of headspace gas for isotopolog analysis using a gas-tight syringe, the activity of AOM was determined in the remaining slurry. For this purpose, each of the remaining slurries was subsampled into duplicate 12-ml glass crimp vials without headspace. AOM rates were determined by injecting 20 μl of ^14^C methane tracer (dissolved in Milli-Q water, activity 0.7 kilobecquerel, specific activity 185 megabecquerel mmol^−1^) to each vial. The vials were incubated in the dark for 2 days at the same temperatures applied to the stock slurries (20°C). To terminate AOM activity, samples were transferred to 50-ml glass vials filled with 20 ml of 5% NaOH. The vials were sealed with rubber stoppers immediately and shaken thoroughly. AOM rates were determined by oven combustion and acidification (*38*). Samples were considered active only if the sample value exceeded the killed control mean + (3 × SD of killed controls). If a sample value surpassed this threshold, the killed control mean was subtracted from the sample value. Taking into account the dilution factor of the slurry, rates of both methanogenesis and AOM in slurry incubations were normalized to nanomole methane per cubic centimeter undiluted sediment per day, facilitating comparisons with the rates obtained through ex situ measurements and modeling.

Ex situ rates of methanogenesis and AOM in sediment cores from the salt marsh and thermokarst lake have been previously published (*43*, *72*, *81*). Briefly, rates of methanogenesis, AOM, and sulfate reduction in salt marsh sediments were tracked using ^14^C-labeled methylamine, ^14^C-labeled methane, and ^35^S-labeled sulfate, respectively (*81*). Note that the methylamine-based methanogenesis rate should be considered as the hypothetical minimum rate of methane production since methanogenesis rates of other pathways and other methylated compounds were not determined. In thermokarst lake sediments, methanogenesis rates were quantified through ^14^C-labeled bicarbonate and acetate in short-term incubation and methane concentration in long-term incubation, while AOM rates were determined by ^13^C-labeled methane (*43*, *72*).

Ex situ rates of methanogenesis were not measured for the Santa Barbara and Guaymas Basins. To determine net rates of methane production and consumption, we used one-dimensional reaction-transport modeling with the software PROFILE (*82*). Assuming that the depth profiles of methane concentration represent a quasi-steady state, the PROFILE model partitioned the sediment pile into discrete depth intervals (e.g., one to four zones), each assigned a constant process rate that best replicated the observed concentration profiles. In this context, transport is exclusively assumed to occur through molecular diffusion—a condition we consider realistic in the deep Santa Barbara Basin where the bottom water is generally hypoxic-anoxic, limiting bioirrigation and bioturbation, and the sediment lacks gas bubbles (*83*, *84*). Porewater methane concentrations at the top and bottom of the modeled depth interval served as boundary conditions. Both measured and modeled volumetric rates are expressed in units of nanomoles per cubic centimeter per day.

In diffusion-dominated marine sediments, most of the methanogenesis occurs in the uppermost part of the methanogenic zone, driven by the steep power-law decline in mineralization rates with increasing depth and sediment age (*30*, *31*). Methane is predominantly generated within an upward-directed gradient, driving its diffusion toward the SMTZ. The observed diffusive flux represents most of the methane production throughout the entire sediment column (*31*). Consequently, the measured isotopolog compositions in the Santa Barbara Basin primarily reflect microbial methane produced in situ.

The same modeling approach cannot be applied to the deep subsurface sediments from the Guaymas Basin because the methane concentration data become unreliable at methane partial pressure greater than 3 to 5 bar, and they are affected by outgassing when the drilling cores were retrieved and subsampled (*31*, *85*). The methane concentrations in the sulfate-rich Carpinteria Salt Marsh sediments, which exhibit little variation, do not allow for the effective determination of a net methanogenesis rate (fig. S2).

### Thermodynamic calculations

Molar Gibbs free energies (Δ*G*) of the three methanogenic catabolisms (reactions 1 to 3) listed below were computed using in situ physicochemical data collected down the sampling interval where methane isotopolog compositions were analyzed. The reactions are

$${\text{4H}}_{2\left(\text{aq}\right)}+{\text{CO}}_{2\left(\text{aq}\right)}\to {\text{CH}}_{4\left(\text{aq}\right)}+{\text{2H}}_{2}O_{\left(l\right)}$$ (1)

$${\text{4CH}}_{3}{\text{OH}}_{\left(\text{aq}\right)}\to {\text{3CH}}_{4\left(\text{aq}\right)}+{\text{CO}}_{2\left(\text{aq}\right)}+{\text{2H}}_{2}O_{\left(l\right)}$$ (2)

$$\begin{array}{c} 4\left({\text{CH}}_{3}\right){\text{NH}}_{2\left(\text{aq}\right)}+{{\text{4H}}^{+}}_{\left(\text{aq}\right)}+{\text{2H}}_{2}O_{\left(l\right)}\to {\text{3CH}}_{4\left(\text{aq}\right)}\\ +{\text{CO}}_{2\left(\text{aq}\right)}+{{{\text{4NH}}_{4}}^{+}}_{\left(\text{aq}\right)} \end{array}$$ (3)

The SUPCRT92 software package was used to compute the standard state Gibbs free energy (Δ*G*^0^) under in situ temperature and pressure conditions, with the consideration that all species are assumed to be in aqueous form (*86*). On the basis of previous studies in similar environments, we assume that the sulfate-free deep-sea sediments were dominated by hydrogenotrophic methanogenesis (*47*, *48*), whereas methylotrophic methanogenesis is the primary pathway in the sulfate-rich salt marsh (*38*, *39*). For hydrogenotrophic methanogenesis, CO_2_ concentrations were calculated from alkalinity and pH data (*87*). For methylotrophic methanogenesis, we used methanol and methylamine as examples. The concentrations of methanol and methylamine at the Carpinteria Salt Marsh were found to be detectable (>3 μM) but fell below quantification limit (10 μM) (*81*). Consequently, a range of 3 to 10 μM for methanol and methylamine was used in the calculations, along with the measured concentrations of other species (CH_4_, CO_2_, NH_4_^+^, and pH). Activities were determined by multiplying the concentrations of reactants and products by their respective activity coefficients (*88*). Given our assessment of two disproportionation reactions, the values of Δ*G*, where Δ*G* = Δ*G*^0^ + RT ln *Q*, are presented in units of kilojoules per mole-carbon-transferred or kilojoules per mole C, facilitating standardized energetic comparisons (*89*). Note that we did not attempt to calculate Δ*G* values for incubation experiments with AOM inhibitors due to the potential concurrent production of methane through multiple methanogenic pathways.

In addition, we include published isotopolog data on AOM and use the averaged value in our analysis (*54*, *55*). We assume that AOM is analogous to reversed hydrogenotrophic methanogenesis and express the Δ*G* of the environment in terms of the energy available for hydrogenotrophic methanogenesis (Fig. 3B). On the basis of this assumption, Dale *et al.* (*60*) reported a Δ*G* value of −11 kJ mol^−1^ C for AOM in the SMTZ. Consequently, the averaged AOM isotopolog data are presented with a positive Δ*G* value of 11 kJ mol^−1^ C with respect to hydrogenotrophic methanogenesis, allowing comparison with isotopolog data from hydrogenotrophic methanogenesis, albeit with some caveats. We consider the Δ*G* value of 11 kJ mol^−1^ C as a rough estimate, which should be regarded as the hypothetical minimum Δ*G* value.

### Doubly substituted isotopolog measurements and isotope notation

Methane isotopolog abundances in methane gas samples were measured using the Panorama (Nu Instruments) high–mass resolution gas-source IRMS housed at the University of California, Los Angeles. Details surrounding the purification and measurement of methane gas were previously published (*10*) and are briefly summarized here (*32*, *55*). Methane gas samples were purified on a vacuum line interfaced with a GC. Samples were delivered to the vacuum line through a septum by a gas-tight syringe and trapped on silica gel at liquid nitrogen temperature. The helium carrier gas was then used to flush the sample to the GC. Separation was accomplished with a 3-m ^1^/_8_-inch (3.175 mm) outer diameter (OD) stainless steel column packed with 5-Å molecular sieve, followed in series by a 2-m ^1^/_8_-inch (3.175 mm) OD stainless steel column packed with HayeSep D porous polymer. Peaks were identified using an in-line, passive thermal conductivity detector (TCD). Once methane collection was complete, the sample was transferred to an evacuated sample tube filled with silica gel at liquid nitrogen temperature. Methane in this tube was introduced to the inlet of the mass spectrometer where it was warmed to 40°C and expanded into the bellow of the instrument.

The Panorama mass spectrometer was set to a mass resolving power of ~40,000 or greater, allowing the measurement of ion currents for resolved ^12^CH_4_^+^, ^13^CH_4_^+^, ^12^CH_3_D^+^, ^13^CH_3_D^+^, and ^12^CH_2_D_2_^+^. Isotopologs of masses 16 and 17 were measured using Faraday collectors with amplifier resistors of 10^11^ ohm. Both doubly substituted mass-18 isotopologs, ^13^CH_3_D^+^ and ^12^CH_2_D_2_^+^, were measured with an electron multiplier as the axial collector. The measured ratios of these ion currents yield values for bulk ^13^C/^12^C and D/H as well as for both Δ^13^CH_3_D and Δ^12^CH_2_D_2_. The isotopic compositions of carbon and hydrogen are reported as deviations from the carbon and hydrogen reference materials Vienna Pee Dee belemnite (VPDB) and VSMOW. Standard delta notation is used to express the fractional differences in per mil units

δC13=[(C13/C12)sample/(C13/C12)VPDB−1]×1000 (4)

$$\mathrm{\delta D}=\left[{\left(D/H\right)}_{\text{sample}}/{\left(D/H\right)}_{\text{VSMOW}}-1\right]\times 1000$$ (5)

The relative abundances of the two mass-18 isotopologs of methane are reported relative to the stochastic reference frame expressed in per mil using the capital delta notation

ΔC13HD3=[(C13HD3/C12H4)sample/(C13HD3/C12H4)stochastic−1]×1000 (6)

ΔC12H2D2=[(C12H2D2/C12H4)sample/(C12H2D2/C12H4)stochastic−1]×1000 (7)

External precision for δ^13^C, δD, Δ^13^CH_3_D, and Δ^12^CH_2_D_2_ is approximately 0.1, 0.3, 0.3, and 0.7‰, respectively (1σ), based on replicate samples. The relationship between temperature and both Δ^13^CH_3_D and Δ^12^CH_2_D_2_ has been predicted through ab initio calculations and can be expressed by the following equations (*10*)

ΔC13HD3(T)≈1000ln(1+0.0355502/T−433.038/T2+1270210.0/T3–5.94804×108/T4+1.196630×1011/T5–9.07230×1012/T6) (8)

ΔC12H2D2(T)≈1000ln(1+0.183798/T−785.483/T2+1056280.0/T3+9.37307×107/T4–8.919480×1010/T5+9.901730×1012/T6) (9)

where *T* is in Kelvin. Equations 8 and 9 show that Δ^13^CH_3_D and Δ^12^CH_2_D_2_ values are both positive when methane is formed at thermodynamic equilibrium and approach 0‰ at high temperatures (>1000 K).

## Supplementary Materials

### The PDF file includes:

Supplementary Text Figs. S1 to S14 Tables S1 to S4 Legend for dataset S1 References
