# Supplementary material for: Clumped isotopes of methane trace bioenergetics in the environment
Source: Sci Adv. 2025 Jun 25;11(26):eadu1401. doi: 10.1126/sciadv.adu1401 (PMC12190011; doi:10.1126/sciadv.adu1401)
Supplement: Supplementary file 1 — Supplementary Text Figs. S1 to S14 Tables S1 to S4 Legend for dataset S1 References [file sciadv.adu1401_sm.pdf]

Science Advances
AAAS

Supplementary Materials for

Clumped isotopes of methane trace bioenergetics in the environment

Jiarui Liu *et al.*

Corresponding author: Jiarui Liu, jiaruiliu@ucla.edu; Edward D. Young, eyoung@epss.ucla.edu;
Tina Treude, ttreude@g.ucla.edu

Sci. Adv. **11**, eadu1401 (2025)
DOI: 10.1126/sciadv.adu1401

The PDF file includes:

Supplementary Text
Figs. S1 to S14
Tables S1 to S4
Legend for dataset S1
References

Other Supplementary Material for this manuscript includes the following:

Dataset S1

Supplementary Text

Site description and sampling procedures

Carpinteria Salt Marsh Reserve

The Carpinteria Salt Marsh Reserve (CSMR) is an estuarine wetland located on the south coast of Santa Barbara County in southern California, United States. Freshwater runoff flows into the marsh through six drainage channels along the northern boundary, while tidal waters enter via an inlet that is open to the Santa Barbara Channel at the southern boundary (*90*). Krause and coauthors (*81*) collected sediment cores across the main channel along the service road and measured rates of methylotrophic methanogenesis and AOM using ^{14}C-radiotracers. Guided by these results, we selected two brackish sites located in close proximity to freshwater runoff (table S1), where AOM rates were found to be less than 5% of the rates of methylotrophic methanogenesis in the upper 5 cm (fig. S2). The organic matter in the surface sediment (0–5 cm) was relatively young, spanning days to years, as evidenced by the recent algae blooms buried within the surface layers (*68*). Sediment in the upper 5 cm of brackish sites 1 and 2 had sulfate concentration of ca. 20 and 26 mM, respectively. We collected sediment from the upper 5 cm at each site in 2022. The sediment was transferred to borosilicate bottles without headspace, sealed with black butyl rubber stoppers, and stored at 4 °C for in vitro slurry incubation. We also collected on-site methane dissolved in sediment for isotopologue analysis. Due to the low methane concentrations (12–20 μM) at the two sites, we modified 3.8-L glass Mason jars with screw plastic lids and airtight silicone gaskets. A hole was drilled through the plastic lid to install a blue butyl rubber stopper for gas sampling. In the field, we transferred ~2500 cm^3 of sediment to each jar. Subsequently, we introduced ~500 g of solid NaCl and ~800 ml of saturated NaCl solution immediately before sealing the lid, ensuring an air headspace of ~250 ml. The jars were vigorously shaken and stored at 20 ºC for one week before transferring the headspace to evacuated 160-ml crimp vials.

Alaska thermokarst lake

Big Trail Lake is located 8 km north of Fairbanks, Alaska, in the Goldstream Valley (table S1). It sits at the confluence of Goldstream Creek and Big Eldorado Creek, approximately 4 km west of Goldstream Mine in Fox. Big Trail Lake is an actively expanding thermokarst lake, currently covering an area of about 40,000 m^2, with average and maximum depths of 1.2 m and 4.1 m, respectively. The lake is notable for its history of thermokarst activity, including vigorous methane seeps (*91*). Aerial photographic records indicate that the lake formed from permafrost between 1949 and 1967 (*46*). Consequently, the organic matter in the lake has undergone degradation for only 50 to 70 years (*43, 46*). Sediment cores were extracted from two sites by percussion coring in March 2020; specifically, Sites D and S were situated in the western and northern portion of the lake, respectively. The lithology between the two sites was identical (*43*). Sediment from a slit layer (unit 1), a peat layer (unit 2), and a dark mud layer (unit 4) at Site S was transferred to glass vials and sealed with blue butyl rubber stoppers for incubation (fig. S3). The vials were immediately flushed with pure nitrogen gas for 3 intervals of 5 minutes, and vigorously shaken between the intervals to remove all traces of methane and oxygen. Porewater

and sediment geochemistry and microbial activity have been previously published (*43, 72*). AOM rates were determined using $^{13}C-CH_4$ and were found to be only around 1% of the methanogenesis rates in the sediment (*72*) (fig. S3B).

Sediment coring established a conduit for the upward migration and ebullition of subsurface methane gas. At Site S in Big Trail Lake, after vibracore coring down to 4 m, three gas samples were obtained from three boreholes using the water displacement method, where glass vials were immersed in the lake water and filled. Subsequently, the vials were inverted, allowing free gas bubbles to be directed into the vials already filled with lake water. Upon displacement of all the lake water by gas, the vials were sealed with blue butyl rubber stoppers while submerged in the water. Similarly, three gas samples were collected from Goldstream Lake, located 1.3 km west of Big Trail Lake (table S1).

Santa Barbara Basin

Located in the California Borderlands, Santa Barbara Basin (SBB) is a coastal basin with a seasonal anoxic water column, reaching a maximum depth of around 600 meters (*83*). The average sedimentation rate in the center of the SBB was estimated to be 0.12 ± 0.02 cm per year (*92*). A gravity core was collected from the depocenter Site NDRO in November 2019 during an expedition aboard R/V *Atlantis* (cruise AT42-19; table S1) (*93*). For methane concentration analysis, 2 cm^3 of sediment was immediately collected using cut-off syringes, and transferred to 12 ml crimp vials containing 5 ml of 2.5% NaOH solution. The vials were then crimp sealed, shaken vigorously, and stored upside down at 4 °C. Around 25 cm^3 of sediment was transferred into argon-flushed 50 ml centrifuge tubes and centrifuged at 4300 $\times$ g for 20 min for porewater analysis. The remaining sediment was transferred to 1-L glass Mason jars for on-site methane isotopologue analysis, following the same sampling procedure as outlined in the salt marsh section. In July 2023, we returned to the same site aboard R/V *Atlantis* (cruise AT50-11) to acquire a higher-resolution geochemical profile, employing the same methodology used in 2019. Furthermore, 6 cm^3 of sediment was transferred to 12 ml glass vials for the analysis of hydrogen concentration. We note that part of the soft surface sediment was lost during both gravity core retrievals. To determine the original sediment depths, we aligned our porewater sulfate data with previously published sulfate profiles from multi- and gravity cores in the depocenter of the basin (*84*).

Guaymas Basin

Guaymas Basin, located in the Gulf of California, is a young marginal rift basin between the western margin of mainland Mexico and the Baja California Peninsula. It is characterized by active seafloor spreading, steep geothermal gradients, and rapid deposition of organic-rich sediments (*94, 95*). During the International Ocean Discovery Program (IODP) Expedition 385 in fall 2019, drilling activities targeted organic-rich sediments and intruded sills, covering an extensive area within the basin (*85*). We focused on two sites with relatively low and high geothermal gradients (table S1 and fig. S13). Site U1550 is situated in the axial graben of the northern spreading segment of the Guaymas Basin. It is characterized by a relatively low

geothermal gradient of 135 °C/km and an average sedimentation rate greater than 69.2 cm/ka. Site U1547 is located about 27 km northwest of the axial graben in the northern Guaymas Basin, within a circular, bowl-shaped hydrothermal mound called Ringvent, that rises approximately 20 m above the seafloor and exhibits a maximum diameter of around 800 m (*96*). It is characterized by a notably high geothermal gradient of 529 °C/km (measured at Hole U1547B) and an average sedimentation rate exceeding 52.4 cm/ka. Additional details about the two drilling sites and their geochemistry have been previously published (*85*).

Three types of methane gas were collected from sediments at depths of 94–204 m and 82–166 m below the seafloor at Sites U1550 and U1547, respectively. Firstly, headspace samples were collected immediately upon retrieval of sediment cores on the core receiving platform. Two replicates of ~5 cm^3 wet sediment were placed in 20 ml glass vials containing 5 ml of 1 M NaOH solution. The vials were capped with a polytetrafluoroethylene/silicone septum and crimp sealed. Following vortexing, the sealed vials were stored upside down at 4 °C. Secondly, the expansion of entrapped gases can create voids in the sediment. To collect void gas samples, a specialized piercing tool was used to puncture the core liner, enabling the gas to expand into a gas-tight syringe connected to the tool. The obtained sample was then transferred to crimp-capped headspace vials pre-filled with saturated NaCl solution. Thirdly, during hard rock coring, a small (5–15 cm long) whole-round sample of subsurface igneous rock was placed into a trilaminated foil barrier bag and immediately sealed. These samples were incubated for at least 24 h at 70 °C to stimulate degassing. Bags were then sampled using a gas-tight syringe through a polytetrafluoroethylene/silicone septum to quantitatively extract gas. This gas, termed rock incubation, was subsampled into crimped septa vials pre-filled with saturated NaCl solution.

In addition to the drilling samples, we obtained surface sediments from a neighboring site characterized by active methane bubbling (table S1). Push cores were collected by the remotely operated vehicle *Jason* aboard R/V *Roger Revelle* (cruise RR2107) in November 2021. The top 20 cm sediment was transferred to 500 ml borosilicate bottles without headspace, sealed with black butyl rubber stoppers, and stored at 4 °C for in vitro slurry incubation.

Kinetic driving force of AOM

We calculated the kinetic driving force of AOM ($F_{K,AOM}$) to further constrain the depth of potential AOM activity in the Santa Barbara Basin (fig. S6C). The $F_{K,AOM}$ for both sulfate and methane was computed using the general Michaelis–Menten model, governing substrate uptake by microorganisms, with the following equation (*97*):

$$F_{K,AOM} = ([CH_4] / (K_{CH4} + [CH_4])) \times ([SO_4^{2-}] / (K_{SO42-} + [SO_4^{2-}])). \quad (10)$$

Here, $[CH_4]$ represents the methane concentration, $[SO_4^{2-}]$ is the sulfate concentration, and K_{CH4} and K_{SO42-} are half-saturation constants (K_M) for methane and sulfate, respectively. Laboratory experiments suggest that K_M values for methane during sulfate-dependent AOM are in the mM range, while those for sulfate are in the sub-mM range (*98, 99*). Therefore, we adopted a K_{CH4} of 10 mM and a K_{SO42-} of 0.1 mM for our calculations (*100*). The $F_{K,AOM}$ values were normalized to the highest $F_{K,AOM}$ in a given core, yielding dimensionless values ranging from 0 (complete kinetic inhibition) to 1 (no inhibition).

Modeling of methanogenesis and anaerobic oxidation of methane

As of now, modeling efforts in methanogenesis have predominantly concentrated on the hydrogenotrophic pathway. This focus arises from the fact that methane generated through acetoclastic and methylotrophic methanogenesis inherits clumping signals from precursor organic substrates, the analysis of which has only recently been developed (*40*). We incorporated metabolic-isotopic and isotopologue flow network models from two recent studies on hydrogenotrophic methanogenesis into our dataset without modification (*35, 36*). Therefore, the modeling output is intended solely to depict a subset of our dataset, encompassing Santa Barbara and Guaymas Basin samples, on-site methane from the subsurface of Alaska thermokarst lakes, and Carpinteria salt marsh incubations with an H_2 headspace.

Further, we used a closed-system steady-state model to comprehend the concurrent methanogenesis and AOM in the incubation of Guaymas Basin slurry (*55, 101*). In this model, the time-dependent evolution of the moles of an isotopologue of methane can be described in terms of a constant rate of production and a rate of oxidation that behaves as a first-order reaction with an invariable rate constant. In this simplest model, where the balance is only between production and consumption, the system can be described as:

$$\frac{dn_i}{dt} = E_i - k_i n_i, \quad (11)$$

where n_i is the moles of the isotopic species of interest, E_i is the source term for i (e.g., rate of methanogenesis), and k_i is the rate constant for the sink i (i.e., the rate constant for oxidation). The solution to Eq. (11) yields:

$$n_i(t) = n_i^0 e^{-k_i t} + \frac{E_i}{k_i}\left(1 - e^{-k_i t}\right), \quad (12)$$

where n_i^0 is the initial moles of i. The moles of species *i* at steady state is obtained by evaluation Eq. (12) where t → ∞, and thus $e^{-k_i t} \rightarrow 0$, yielding:

$$n_i(t \sim \infty) = \frac{E_i}{k_i}. \quad (13)$$

Considering *i* to be the major isotopologue, the steady-state amount of methane is controlled by the ratio of the production rate and the rate constant for oxidation. The steady-state ratio of two isotopologues can therefore be expressed as:

$$\frac{n_j(t \sim \infty)}{n_i(t \sim \infty)} = \frac{E_j}{E_i}\frac{k_i}{k_j} \quad (14)$$

where *n* is the moles of the two isotopologues *i* and *j*. The steady-state isotopologue ratio depends on the isotopologue ratio of the methane produced (E_j/E_i) and the reciprocal of the fractionation due to oxidation (k_i/k_j), independent of the absolute rates. The ratio of the steady-state moles to initial moles of methane, $(E/k)/n_0$, was determined by assessing the changes in methane concentrations within the bottles. Methane accumulation was observed in the Guaymas Basin slurry without initial methane, while methane consumption was noted in the slurry containing a methane headspace. These observations indicate net methanogenesis and net AOM occurring in the respective incubations. The ratio $(E/k)/n_0$ was set to 500 and 0.2, respectively, based on measured methane concentrations. We set the clumped isotopologue fractionation factors $^{13D}\gamma$ and $^{D2}\gamma$ to be 0.985 and 0.912, respectively, as defined and reported in (*55*). The bulk isotope fractionation factors, $^{13}\alpha$ and $^{D}\alpha$, were set to be 0.962 and 0.738, respectively, through the fitting of measured data. These values fall within the range established by previous studies (*102*). The steady-state clumped isotopologue compositions are solely controlled by $^{13D}\gamma$ and $^{D2}\gamma$, while $^{13}\alpha$, $^{D}\alpha$ and $(E/k)/n_0$ only contribute to shaping the curvature of the trajectory toward the steady state (*55*). We note that in the slurry containing a methane headspace, the source term includes both the tank methane added at the beginning of the incubation and the newly produced methane by methanogens throughout the incubation. We simplify the system with a single effective source term, *E*, characterized by isotopologue compositions predominantly from the tank methane, albeit with caveats.

Methanogenic pathways in Carpinteria Salt Marsh sediments

In sulfate-depleted marine sediments, methane is generated through two primary pathways: the reduction of CO_2 with H_2 as the electron donor (hydrogenotrophic methanogenesis) and the disproportionation of acetate (acetoclastic methanogenesis) (*103, 104*). Methanogens are not able to effectively compete with sulfate-reducing bacteria for common substrates, especially H_2 and acetate (*48*). The free energy available from sulfate reduction exceeds that of methane production in the sulfate zone (*66*). While hydrogenotrophic and acetoclastic methanogenesis are largely inhibited in the sulfate-reducing zone, methylotrophic methanogenesis is known to occur in this zone. It is well established that methylated compounds, such as methylamine and methanol, are important non-competitive substrates for methanogenesis in the presence of sulfate reduction (*39, 105, 106*).

As a result, methylotrophic methanogenesis was suggested to be the predominant pathway for methane production in the sulfate-reducing sediments within the Carpinteria Salt Marsh (*38, 81*). Indeed, elevated methanogenesis rates, determined using ^{14}C-methylamine radiotracer, were observed in the upper 5 cm of sediment, coinciding with the peak in sulfate reduction rates (fig. S2). We suggest that the contributions of hydrogenotrophic and acetoclastic methanogenesis are negligible (*105, 107*). It is important to acknowledge that the methanogenesis rates derived from ^{14}C-methylamine only represent a fraction of the methylotrophic methanogenesis rates, likely underestimating the gross methanogenesis rates since methanogenesis from other methylated compounds is not taken into account.

In investigating the isotopologue effects of the three methanogenic pathways, we introduced H_2, acetate and methylamine to slurries incubations of the salt marsh sediments. Incubations with methylamine exhibited the highest methanogenesis rates, reaching approximately 3000 nmol cm^{-3} d^{-1}, consistent with the ongoing natural methylotrophic methanogenesis. Methanogenesis rates in H_2 incubations varied from 70 to 197 nmol cm^{-3} d^{-1}. We calculated a ΔG of −113.5 kJ mol^{-1} C for hydrogenotrophic methanogenesis in the incubation, consistent with values reported in typical laboratory culture experiments (*24*). Methanogenesis rates in acetate incubations were two orders of magnitude lower than those in H_2 incubations, indicating the minor importance of acetoclastic methanogenesis in the investigated salt marsh sediments. The results suggest that either the sediment lacks the requisite microorganisms capable of using acetate for methanogenesis, or the acetate was used for other processes. Therefore, we argue that introducing molybdate and H_2S to sediment slurries mostly stimulates hydrogenotrophic but not acetoclastic methanogenesis, complementing the concurrently occurring methylotrophic methanogenesis. Methane produced in molybdate incubations aligns with the mixing lines between the methylotrophic and hydrogenotrophic end-members (fig. S4), providing additional support for our interpretation.

Methanogenic pathways in Alaska lake sediments

Based on short-term radiotracer incubations, approximately 43% of methanogenesis in unit 2 occurred through the hydrogenotrophic pathway, with the remaining portion occurring via the acetoclastic pathway (fig. S3C). In unit 4 at the sediment surface, all methanogenesis followed

the acetoclastic pathway (fig. S3C). However, the isotopologue compositions of methane from the two units exhibit only minor differences (fig. S5). Two possible explanations arise: first, isotopologue values may not be entirely indicative of the metabolic pathways of methanogenesis in certain natural environments (*33*). Second, the occurrence of methylotrophic methanogenesis in the lake sediments cannot be ruled out, as the short-term incubation did not consider the role of methyl compounds (*43*). Collectively, the thermokarst lake sediments demonstrate a transition from acetoclastic to hydrogenotrophic methanogenesis downcore, with potential involvement of methylotrophic methanogenesis. Due to the intricate biogeochemical cycling of methane at this site, calculations for Gibbs free energy were not pursued.

<u>Evaluation of Gibbs free energies at the marine sites</u>

In marine sediments, methanogenesis starts in the absence of sulfate at a subsurface depth where the buried organic matter has become notably recalcitrant to microbial degradation. As a result, it is well established that hydrogenotrophic methanogenesis predominates in marine sediments (*47, 48, 108, 109*). To assess the Gibbs free energies at the two marine settings, porewater H_2 concentrations were determined using two distinct methods. H_2 concentrations were measured using a headspace equilibration technique for the Santa Barbara Basin sediment (*75*), whereas an extraction method was applied to the Guaymas Basin sediment (*61, 85*). Each method has its advantages and drawbacks, but the extraction method was found to yield higher H_2 concentrations compared to the headspace equilibration technique (*61*). Therefore, the ΔG values at the two sites are observed in distinct ranges (table S3). Recognizing that this variation could stem from technique-related challenges, we propose considering the higher range, ranging from −22.6 to −14.7 kJ mol^{-1} C, as hypothetical maximum values. These values imply that hydrogenotrophic methanogenesis was exergonic beneath the SMTZ and was close to the suggested energetic limit of methanogenic archaea, which falls between −10 to −20 kJ mol^{-1} C (*75*). Using the lower range from −36.2 to −25.1 kJ mol^{-1} C as hypothetical minimum values, they are half of the ΔG values calculated for the salt marsh sediments (table S3). This indicates a notable difference in the thermodynamic driving force between salt marsh and deep-sea sediments.

<u>Comparison between energy-rich and energy-limited sites</u>

Age controls on organic matter reactivity are discussed in the main text, and there are additional lines of evidence supporting our choice of study sites.

- Volumetric organic carbon oxidation rate. To estimate the organic carbon oxidation rate within the methanic zone of the two marine basins, we extrapolated measured sulfate reduction rates (SRR) down through the methanic zone, because the intrinsic rate of organic matter mineralization is independent of whether the terminal process is sulfate reduction or methanogenesis (*47*). In the gravity core from the Santa Barbara Basin, the SRR decreased from 3.9 nmol cm^{-3} d^{-1} at 59 cm to 0.3 nmol cm^{-3} d^{-1} at 113 cm depth (*110*). Therefore, the methanogenesis rate below the SMTZ (150 cm depth) must be lower than ~0.3 nmol cm^{-3} d^{-1}, which is independently confirmed by modeling results using the porewater methane profile (0.2

nmol cm^{-3} d^{-1}). Similarly, in drilling cores from the Guaymas Basin, the SRR above the SMTZ was approximately 1 nmol cm^{-3} d^{-1} (*111*). In contrast, we sampled surface sediment (0–5 cm) from the salt marsh for on-site methane isotopologue analysis, where SRR ranged from 115 to 1615 nmol cm^{-3} d^{-1}—two to three orders of magnitude higher than in the deep marine sediments.

- Areal organic carbon oxidation rate. To evaluate the overall reactivity of organic matter throughout the sediment column at each site, we calculated the areal organic carbon oxidation rate by integrating SRR across the sediment column. This approach excludes aerobic respiration and other mineralization pathways, leading to an underestimation of the total oxidation rate at the salt marsh due to high oxygen and nitrate concentrations in the creek water. However, it closely approximates the rate at the depocenter of the Santa Barbara Basin, where oxygen concentrations in the bottom water are depleted (<1 μM). The integrated SRR at the Santa Barbara Basin ranged from 1.7 to 4.1 mmol m^{-2} d^{-1} (*112*), and at the salt marsh, it ranged from 11.0 to 42.2 mmol m^{-2} d^{-1} (*81*). In the non-hydrothermal part of the Guaymas Basin, where our study sites are situated, the integrated SRR was 1.4 mmol m^{-2} d^{-1}, equivalent to 2.8 mmol C_{org} m^{-2} d^{-1}, assuming a stoichiometric ratio of 2 for organic carbon mineralization to sulfate reduction (*113*). The areal rates of aerobic organic mineralization found in most other deep-sea sediments at 2000 m water depth are approximately 1 mmol m^{-2} d^{-1}, which are measured as oxygen uptake rates (*114*). Combining the two values, the areal organic carbon oxidation rate in the Guaymas Basin is on the order of 3.8 mmol m^{-2} d^{-1}. These values are summarized in table S4. We emphasize that these estimations are very rough and are intended solely for comparison purposes.

- Minimal methanogenesis in oligotrophic abyssal sediments. One could argue that the sediments from the Santa Barbara and Guaymas Basins contain more reactive organic carbon compared to those from the abyssal plains beneath ocean gyres. However, methane isotopologue data from strongly energy-limited sediments beyond continental margins are absent, where methanogenesis plays a minimal role. As shown in the map by Egger et al. (*115*), the majority of areas beyond continental margins have no SMTZ, suggesting little to no methanogenesis throughout the sediment column. Therefore, the Santa Barbara and Guaymas Basins are representative of typical marine sediments, making them well-suited to test the hypothesis that energy limitation drives the equilibrium of methane clumped isotopologues.

Co-variation of methanogenic pathways and energy conditions

In natural environments, we acknowledge that the co-variation of methanogenic pathways and energy conditions complicates attributing the degree of isotopic disequilibrium to either energy or methane-producing pathways. This complexity is intrinsic to natural environments, where pathways shift in response to changes in environmental conditions, such as free energy availability, temperature, organic matter composition, and sulfate concentration (*41*). While hydrogenotrophic methanogenesis dominates in the methanic zone of marine sediments, surface freshwater environments often exhibit a mix of hydrogenotrophic and acetoclastic methanogenesis (*41, 43, 47*). In certain freshwater environments, such as wetlands, methanogenesis shifts from the acetoclastic to the hydrogenotrophic pathway with increasing depth, because the buried organic matter becomes less degradable and the microbial communities face increased energy limitations (*45*). This shift underscores that the availability of

free energy is a key control over the dominant methanogenic pathway. In the main text, we use the available ΔG values of hydrogenotrophic methanogenesis to quantitatively address the energy hypothesis.

Comparison of microbial versus non-microbial methane in the Guaymas Basin

Guaymas Basin is characterized by high heat flow and magmatism, along with rapid deposition of organic-rich sediments (*94, 95*). Magmatism, in the form of sill intrusions into sediments, provides transient heat sources that mobilize buried sedimentary organic material, leading to the formation of thermogenic methane and other hydrocarbons (*116*). The sedimentary carbon also serves as potential microbial substrates, fostering the production of microbial methane in sediment with lower temperatures (*117*). To investigate the formation mechanism of methane under varying geothermal gradients, we analyzed the isotopologue compositions of methane collected from two drilling cores within the basin.

At Site U1550, the in-situ temperatures varied from 16 to 31 °C across the sampling depths for methane clumping analysis (fig. S12C). The notably high $C_1/(C_2+C_3)$ ratios, ranging from 630–4022, along with low apparent temperatures derived from $\Delta^{13}CH_3D$ (40–62 °C), suggest that microbial methanogenesis predominantly contributes to methane formation at relatively low temperatures (figs. S12 and S13). The negative ΔG values, approximately −30 kJ mol^{-1} C, further support the occurrence of hydrogenotrophic methanogenesis (table S3). Functional gene sequencing has detected functionally and phylogenetically diverse methanogens at Site U1550, including members of the *Methanosaetaceae*, *Methanosarcinaceae*, *Methanobacteraceae*, and uncultured lineages (*118*). Essential genes for methanogenic archaea are expressed in sediment from this site (*119*). Site U1550, along with other microbial gases characterized by elevated $C_1/(C_2+C_3)$ ratios (fig. S13), is discussed in detail in the main text.

On the contrary, Site U1547 displayed notably higher in-situ temperatures, ranging from 55 to 100 °C (fig. S12F). The $C_1/(C_2+C_3)$ ratios fell within the range of 80 to 142 (fig. S13), below the typical microbial methane threshold of ~200 (*7*). Positive values for ΔG in hydrogenotrophic methanogenesis further exclude microbial contributions from this pathway (table S3). Recent studies highlight that $\Delta^{13}CH_3D$ values of thermogenic methane gases align with thermodynamic equilibrium at their formation temperatures, whereas a deficit in $\Delta^{12}CH_2D_2$ can occur due to a combinatorial effect (*13, 18, 120*). Indeed, clumped isotope analysis of methane unveiled varying levels of disequilibrium in $\Delta^{12}CH_2D_2$ values (fig. S14). This departure from equilibrium is most prominent at low thermal maturities, and $\Delta^{12}CH_2D_2$ tends to approach equilibrium with increasing maturity (*13*). The apparent temperatures, based on $\Delta^{13}CH_3D$ values, varied between 141 and 239 °C, falling within the thermogenic gas window (*13*). Notably, these temperatures were higher than the in-situ temperatures at the gas sampling location. This discrepancy suggests that the gases likely originated at greater depths, where methane reached isotopologue equilibrium with the ambient temperature, and subsequently migrated upward to sediment with lower temperatures, where isotope exchange proceeds at a slower rate (*121*). In summary, methane collected below 80 m below the seafloor at Site U1547 is consistent with a thermogenic origin, although we cannot entirely rule out the possibility of minor mixing with microbial methane at shallower depths (*89*). At shallower depths above 74 m below the seafloor, functional

gene sequencing at Site U1547 detected members of the *Methanosarcinaceae*, *Methanomicrobiaceae*, and the hyperthermophilic *Methanocaldococcaceae* (*118*). Within the same depth horizon, essential genes for methanogenesis remain expressed in Site U1547 sediment (*119*).

Supplementary Figures S1–S14

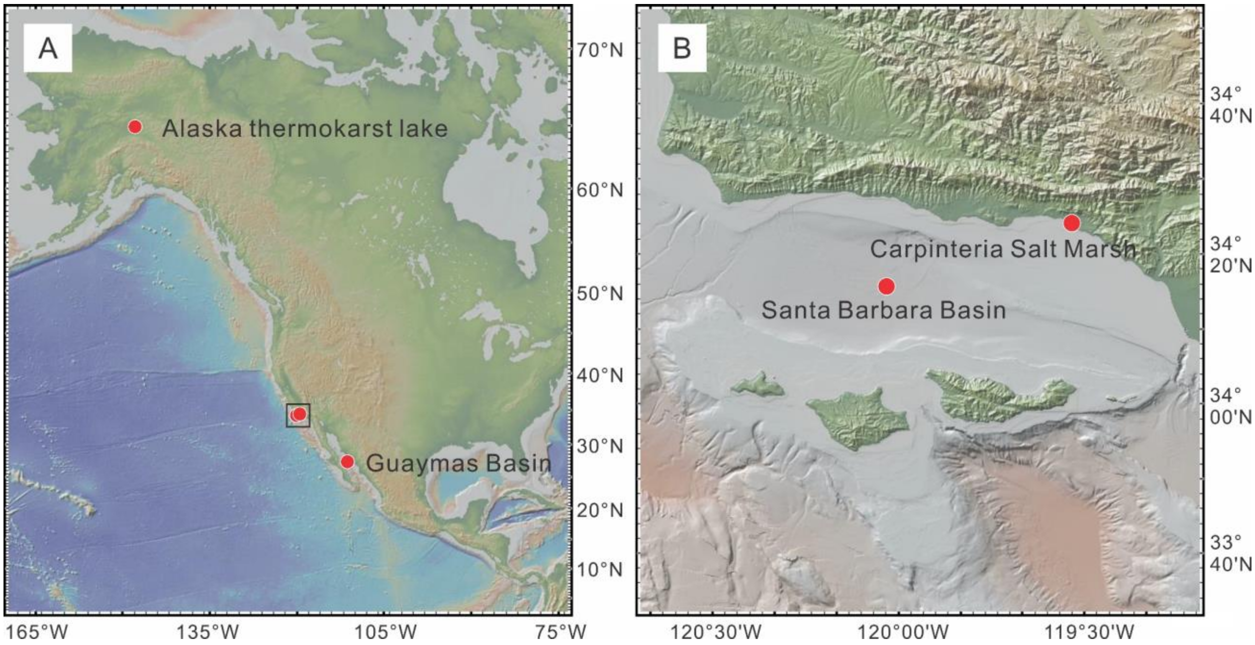

Fig. S1.

Maps with sampling sites. Black box in map A shows location of map B. The maps were generated via GeoMapApp.

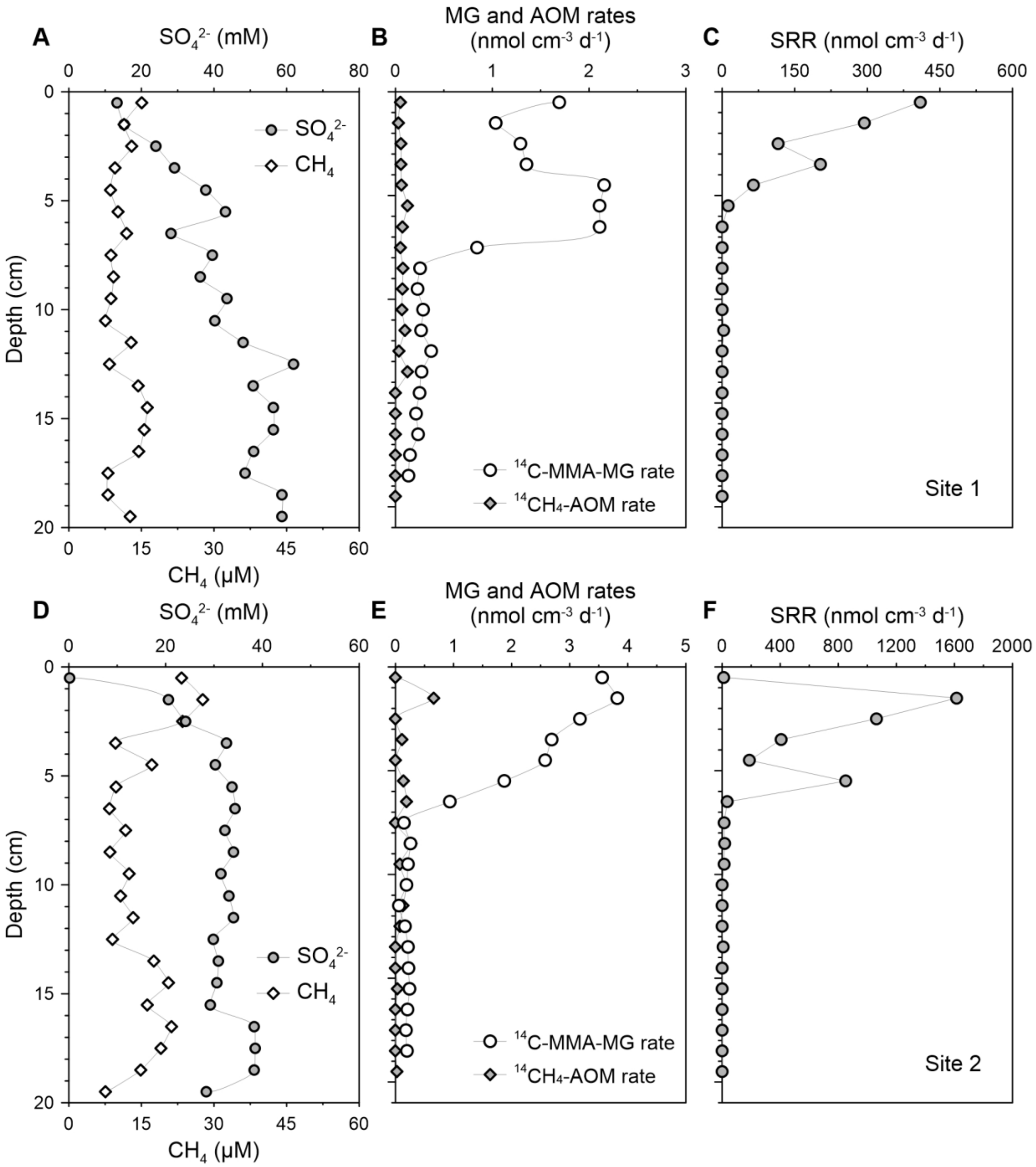

Fig. S2.

Depth profiles of biogeochemical parameters in sediments of two sites within the Carpinteria Salt Marsh. (A, D) Porewater sulfate and methane concentrations. (B, E) Rates of monomethylamine-based methanogenesis (MMA-MG) and AOM determined from direct injection of ^{14}C-MMA and ^{14}C-CH_4, respectively. (C, F) Sulfate reduction rates (SRR) determined from direct injection of ^{35}S-sulfate. The top and bottom panels depict Sites 1 and 2, respectively. Data were adopted from (*81*).

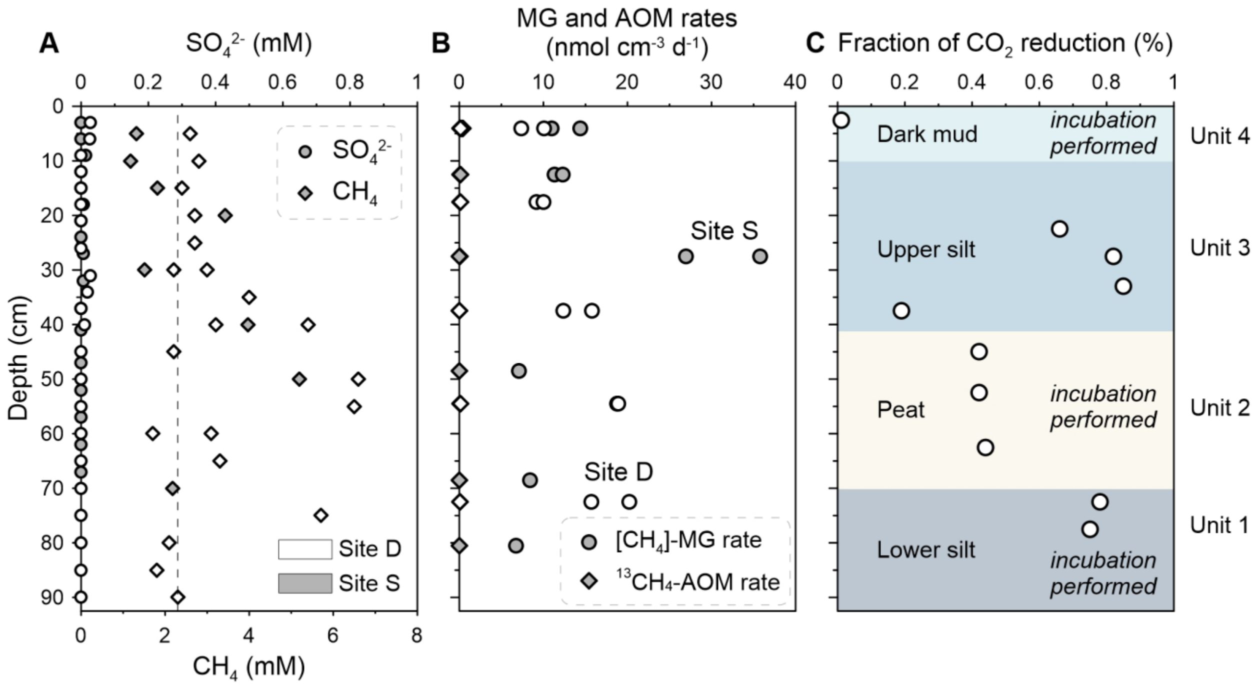

Fig. S3.

Depth profiles of biogeochemical parameters in sediments of two sites within the Big Trail Lake, a thermokarst lake located in Alaska. (A) Porewater sulfate and methane concentrations. The dashed line denotes the concentration of methane saturation at ~4 °C. (B) Rates of methanogenesis and AOM determined from long-term methane concentration analysis and incubation using ^{13}C-CH_4, respectively. (C) The fraction of hydrogenotrophic methanogenesis relative to the total methane production (hydrogenotrophic and acetoclastic methanogenesis) determined from short-term incubation using ^{14}C-labeled bicarbonate and acetate. Data were adopted from (*43, 72*), in which lithological units shown in the background were described.

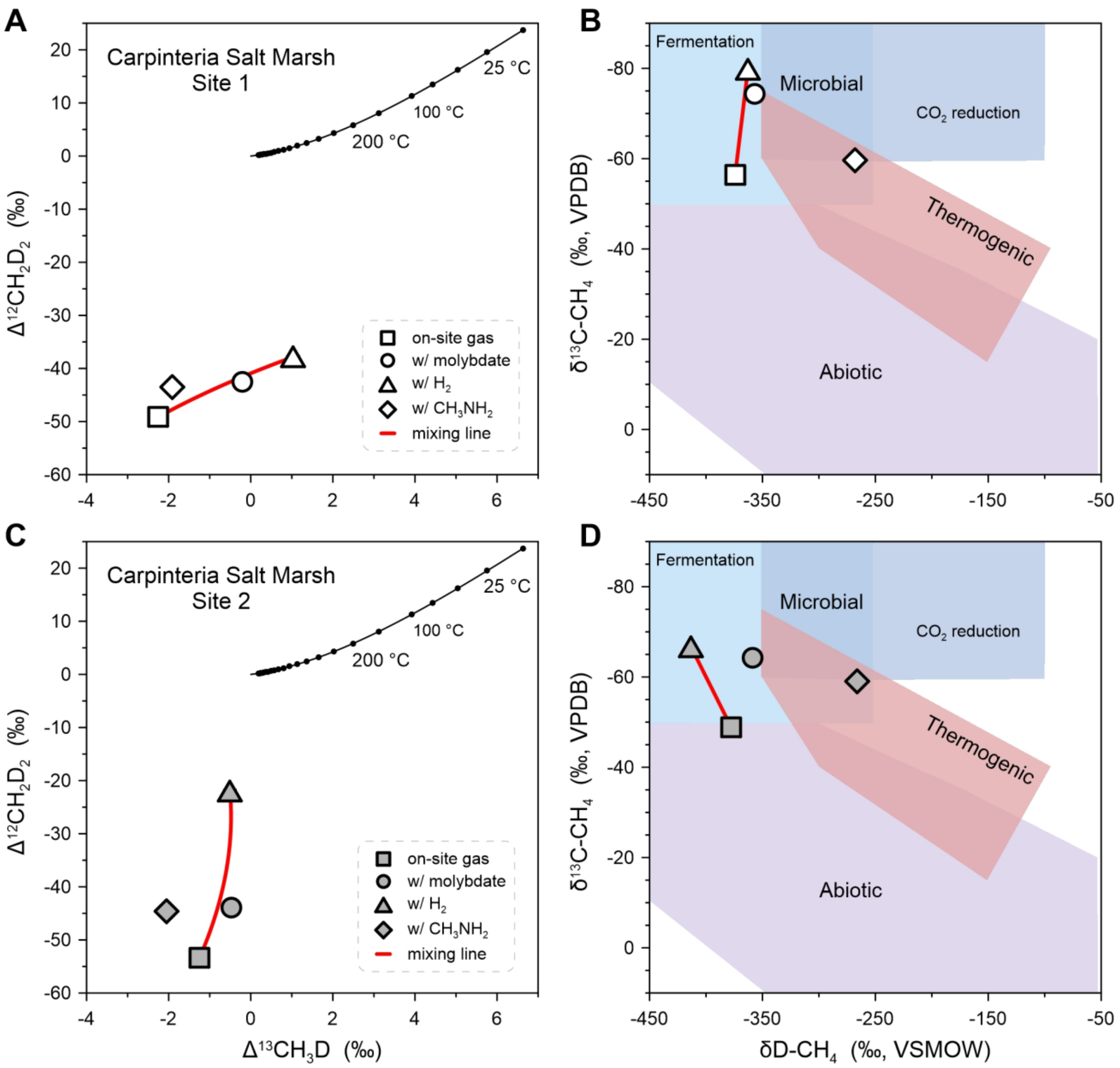

Fig. S4.

Isotopologue compositions of methane samples from the Carpinteria Salt Marsh. (A, C) $\Delta^{13}CH_3D$ plotted against $\Delta^{12}CH_2D_2$. The solid black curves depict theoretical thermodynamic equilibrium abundances of methane isotopologues, along with corresponding temperatures. The red curves represent mixing lines between the two methanogenic end-members, the methylotrophic and hydrogenotrophic pathways. (B, D) $\delta^{13}C$ plotted against δD. The genetic fields for methane sources follow (*5*, *7*). The top and bottom panels depict Sites 1 and 2, respectively.

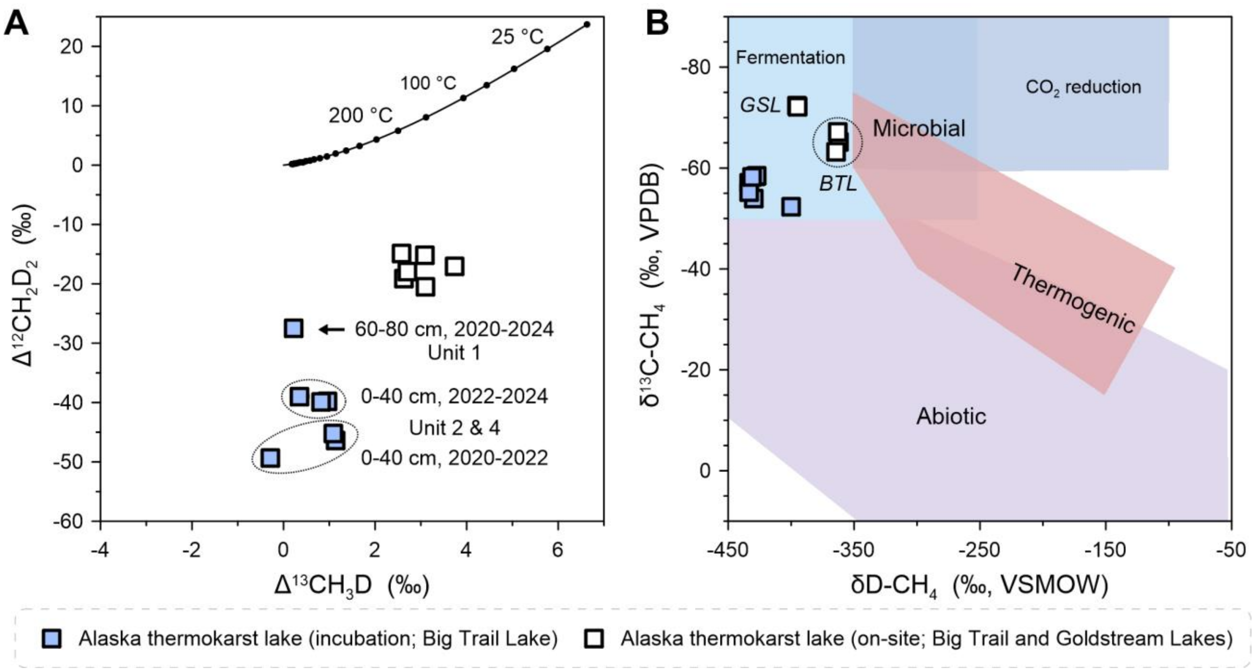

Fig. S5.

Isotopologue compositions of methane samples from the Alaska thermokarst Lakes. Lithological units are shown in fig. S3. The initial incubations for units 2 and 4 extended from 2020 to 2022. The samples from these units underwent argon reflushing in 2022 and were subsequently incubated for another two years. BTL and GSL denote Big Trail Lake and Goldstream Lake, respectively.

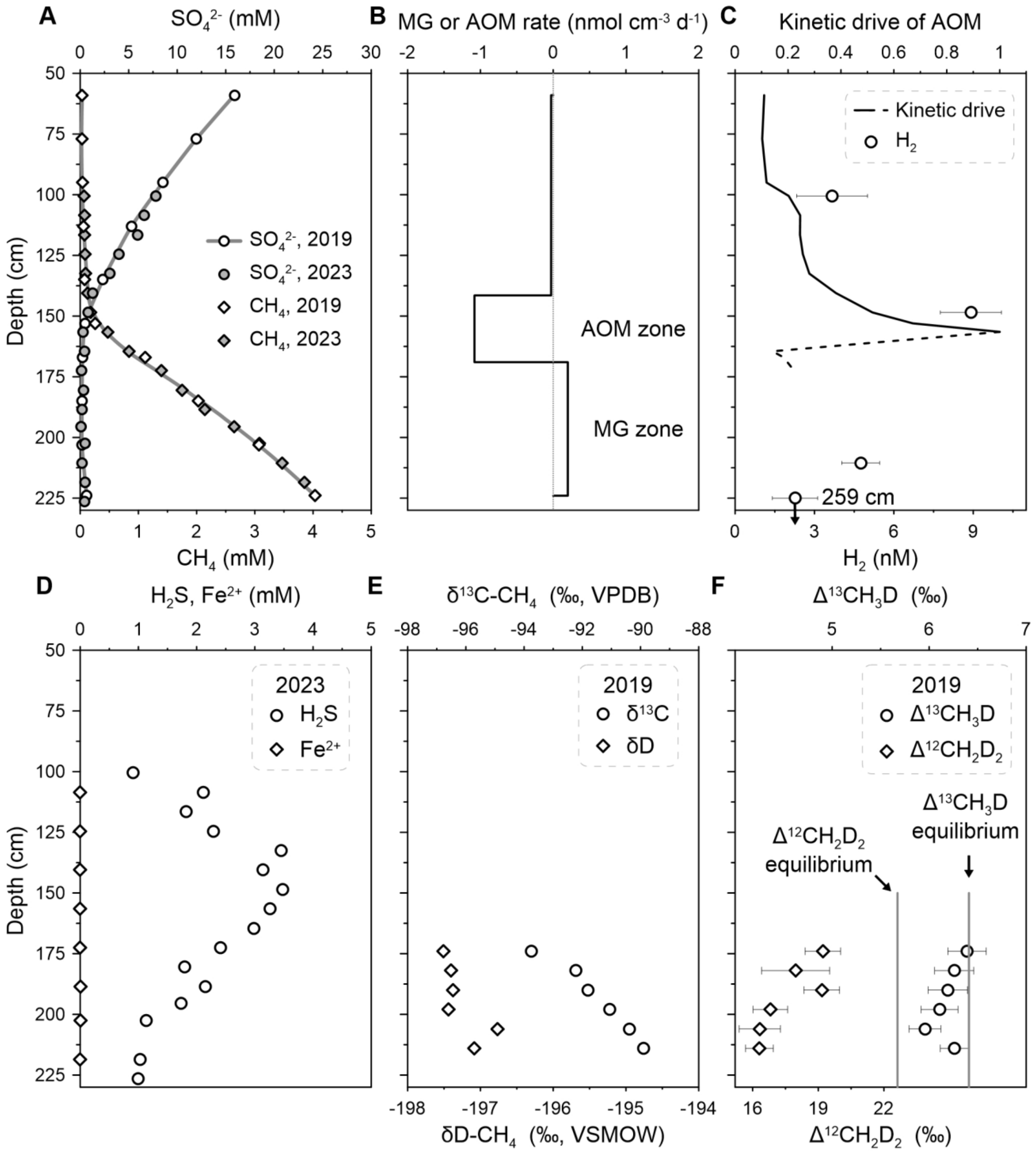

Fig. S6.

Depth profiles of biogeochemical parameters in sediment of Site NDRO within the Santa Barbara Basin. (A) Porewater sulfate and methane concentrations. The grey line through methane data represents the model fit to the methane concentration data. (B) Modeled rates of net methanogenesis (MG) and AOM. (C) Kinetic drive of AOM and porewater hydrogen concentration. The solid line was derived from measured sulfate and methane concentrations,

while the dashed line was calculated by assuming sulfate concentrations of 0.01 mM below the SMTZ (*49*). (D) Porewater aqueous sulfide and dissolved iron(II) concentrations. (E) Bulk isotopic compositions of methane. (F) Clumped isotopologue compositions of methane. The solid grey lines denote equilibrium isotopologue compositions for the in-situ temperature of 6 °C. Error bars are one standard error.

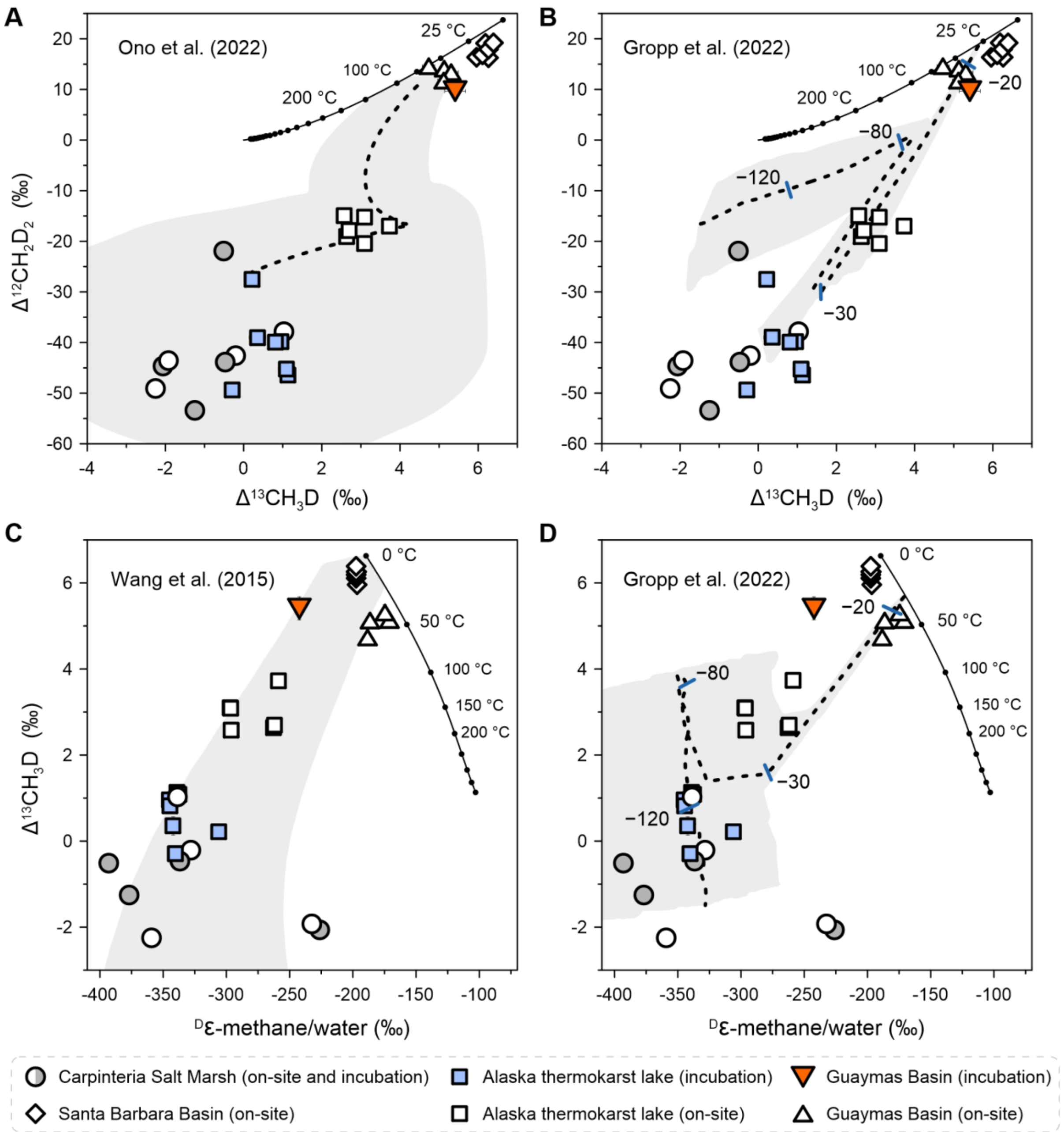

Fig. S7.

Model-observation comparison plotted in $\Delta^{13}CH_3D$ versus $\Delta^{12}CH_2D_2$ (A–B) and $^{D}\varepsilon$-methane/water versus $\Delta^{13}CH_3D$ (C–D). The modeling results were redrawn onto the data presented in Figs. 1 and 2 (*9, 35, 36*). Note that the model outputs are only applicable to hydrogenotrophic methanogenesis. The dashed black lines show the median of the simulations. Data from the Carpinteria Salt Marsh are represented by circles, with site 1 shown in white and site 2 in grey. Panels B and D depict simulations without the methylene-H4MPT dehydrogenase enzyme, with tick marks at ΔG_{net} values of −20, −30, −80, and −120 kJ mol^{-1}.

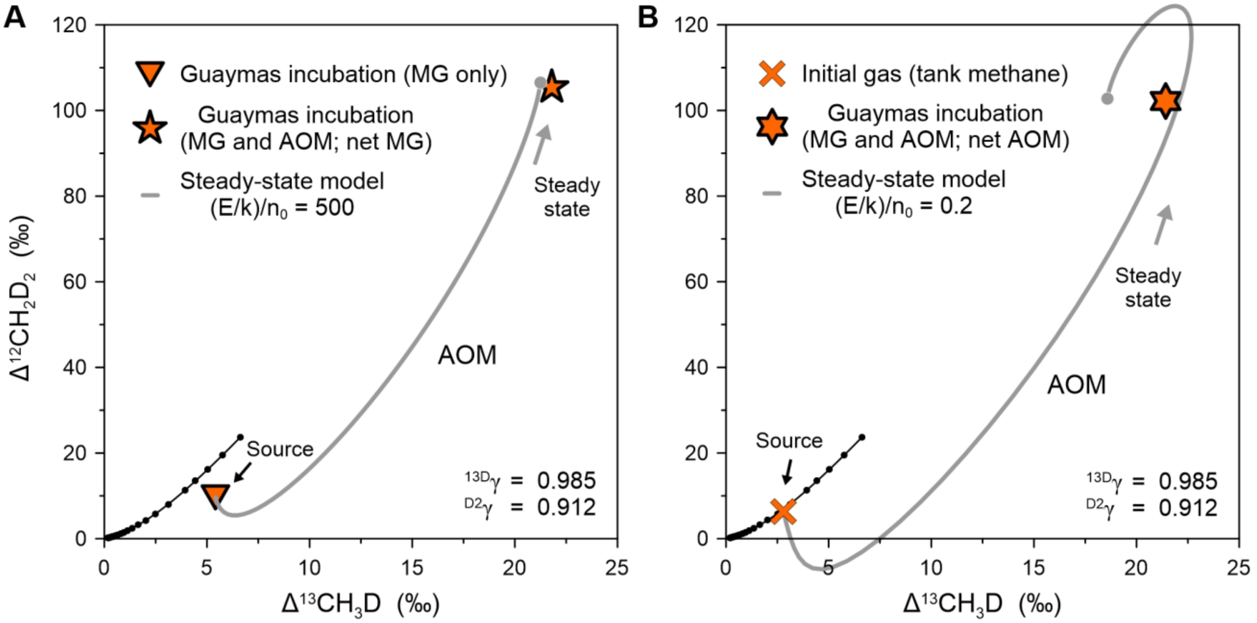

Fig. S8.

Isotopologue compositions of methane samples from the incubations of the Guaymas Basin slurries. In panel A, the orange triangle represents the methane source produced by methanogenesis, while the orange star depicts the resultant methane, reflecting concurrent methanogenesis and AOM leading to net methanogenesis. The incubation began with no methane present in the headspace. In panel B, the cross symbol denotes the methane source of tank methane, while the orange hexagram signifies the resulting methane, reflecting concurrent methanogenesis and AOM leading to net AOM. The incubation started with tank methane (200 kPa) in the headspace. Grey lines depict simulation outputs of the closed-system steady-state model. The clumped isotopologue fractionation factors, adopted from (*55*), and the ratio of the steady-state moles to initial moles of methane, $(E/k)/n_0$, used in the model, are shown in each panel.

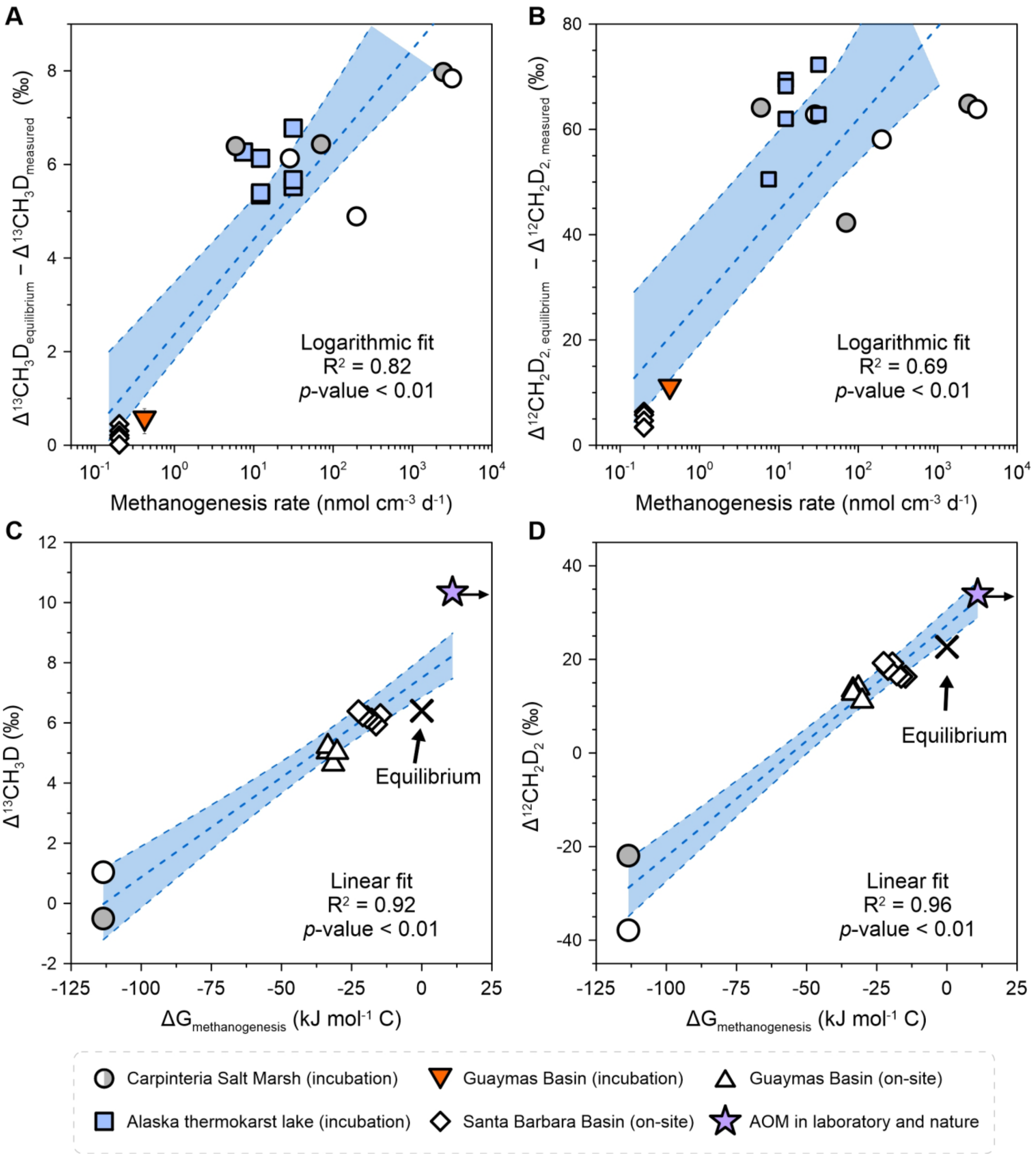

Fig. S9.

Methanogenesis rate (A–B) and Gibbs free energy (C–D) plotted against isotopologue data. Linear regressions are presented with 95% confidence intervals. R-squared and p-values are provided in each panel. Data from the Carpinteria Salt Marsh are represented by circles, with site

1 shown in white and site 2 in grey. Methanogenesis rates of Alaska thermokarst lake incubations were adopted from (*72*). Methanogenesis rates for on-site methane in the Santa Barbara Basin were determined through modeling based on methane concentration profiles, illustrated in fig. S6B. The departures from isotopologue equilibrium in panels A–B are defined as the difference between equilibrium isotopologue compositions at in-situ or incubation temperatures and measured isotopologue compositions. In panels C–D, the published AOM isotopologue data are averaged (*54*, *55*) and presented with a published ΔG value of 11 kJ mol^{-1} C with respect to hydrogenotrophic methanogenesis (*60*), offering a conservative estimate. The X markers in panels C–D denote Gibbs free energy of zero and equilibrium isotopologue compositions at 6 °C. Note that the regression is intended solely to illustrate the statistical relationship between these variables and does not imply a universal relationship.

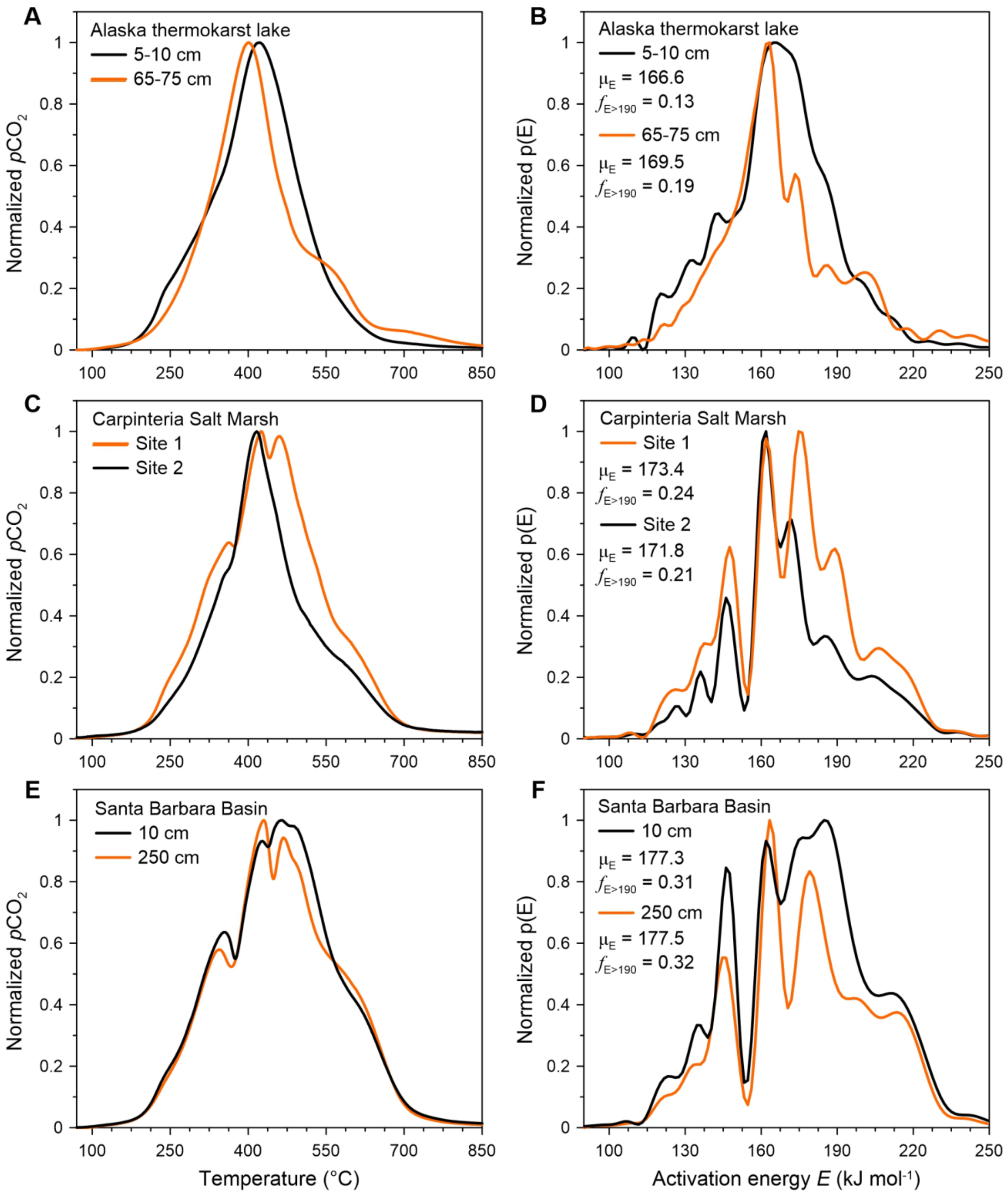

Fig. S10.

Patterns of thermograms and activation energy profiles for the Alaska thermokarst Lake (A–B), the Carpinteria Salt Marsh (C–D), and the Santa Barbara Basin (E–F) sediments. Mean activation energy (μ_E) and fractions of organic carbon with activation energy values greater

than 190 kJ mol^{-1} (f_E>190; more refractory organic carbon) are shown in panels B, D and F. Ramped pyrolysis/oxidation analysis quantifies the thermochemical stability of organic matter and, therefore, determines the proportion of refractory versus labile organic carbon in sediment. We acknowledge that the difference in activation energy distributions of organic carbon between these sites is relatively small, partly because microorganisms can access only a very small fraction of the bulk organic carbon, and much of the higher-energy organic carbon is unlikely to be respired by microorganisms—at least not within any reasonable environmental timescale (*29*, *57*).

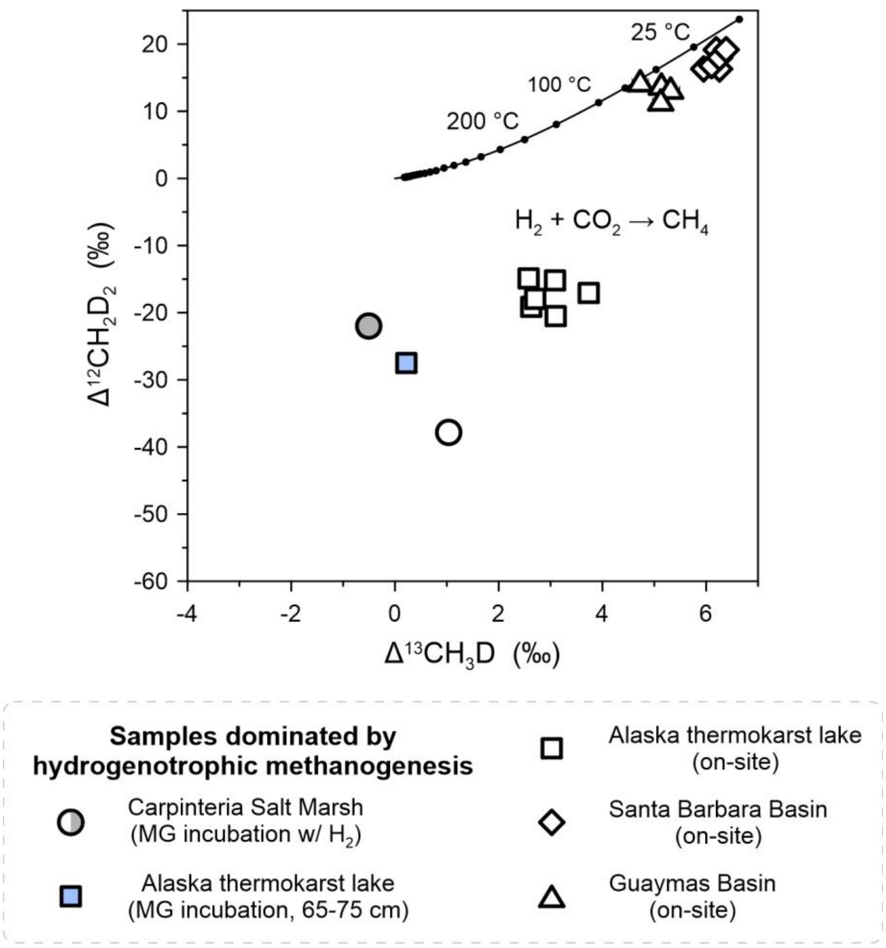

Fig. S11.

Isotopologue compositions of methane samples dominated by hydrogenotrophic methanogenesis in this study.

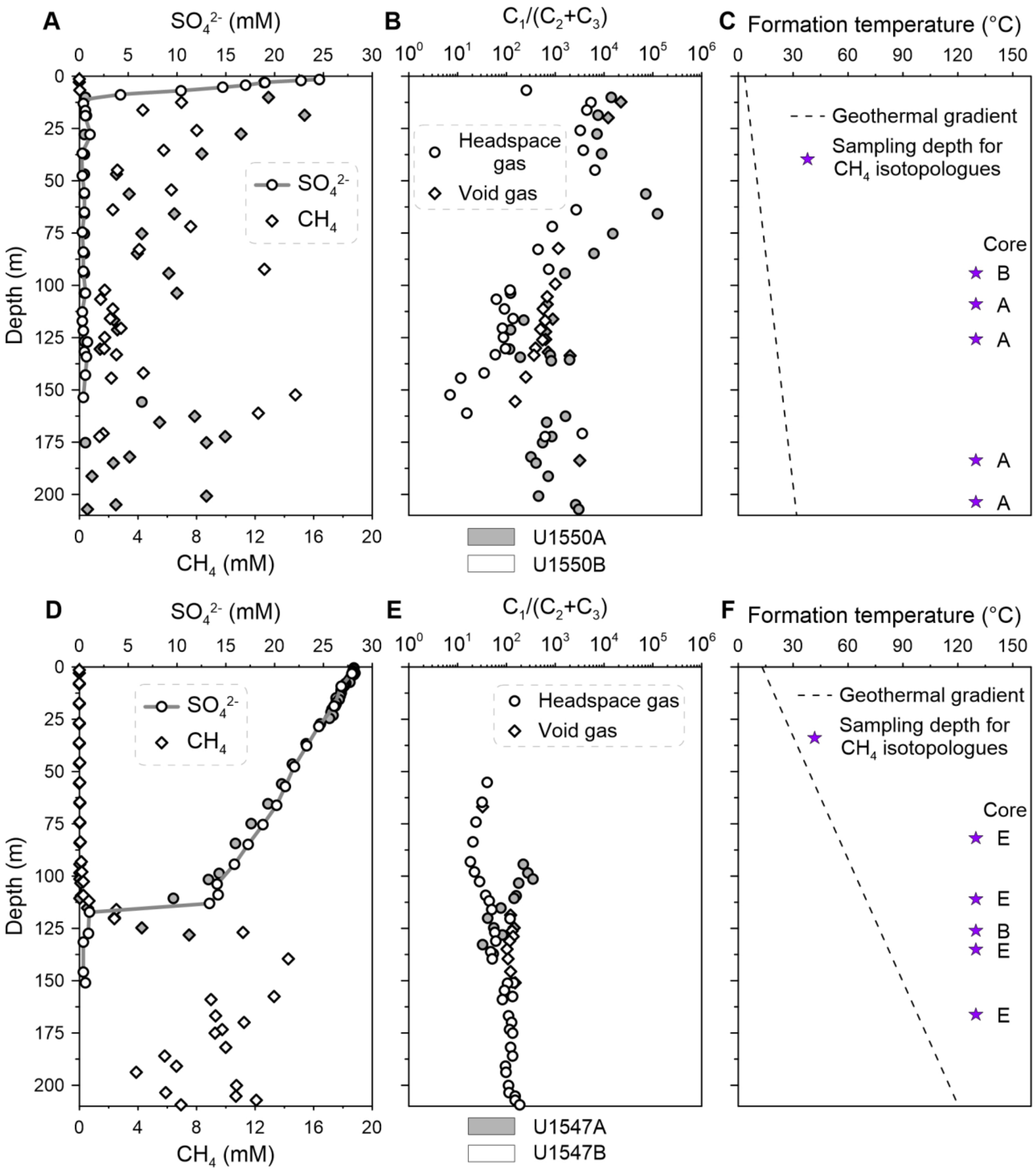

Fig. S12.

Depth profiles of physical and geochemical parameters in sediments of two sites within the Guaymas Basin. (A, D) Porewater sulfate and methane concentrations. (B, E) Ratios of C_1 versus C_2 + C_3. (C, F) Formation temperatures along the drilling cores, accompanied by the depths at which methane samples were analyzed for isotopologue compositions. The top and bottom panels depict Sites U1550 and U1547, respectively. Data were adopted from (*85*).

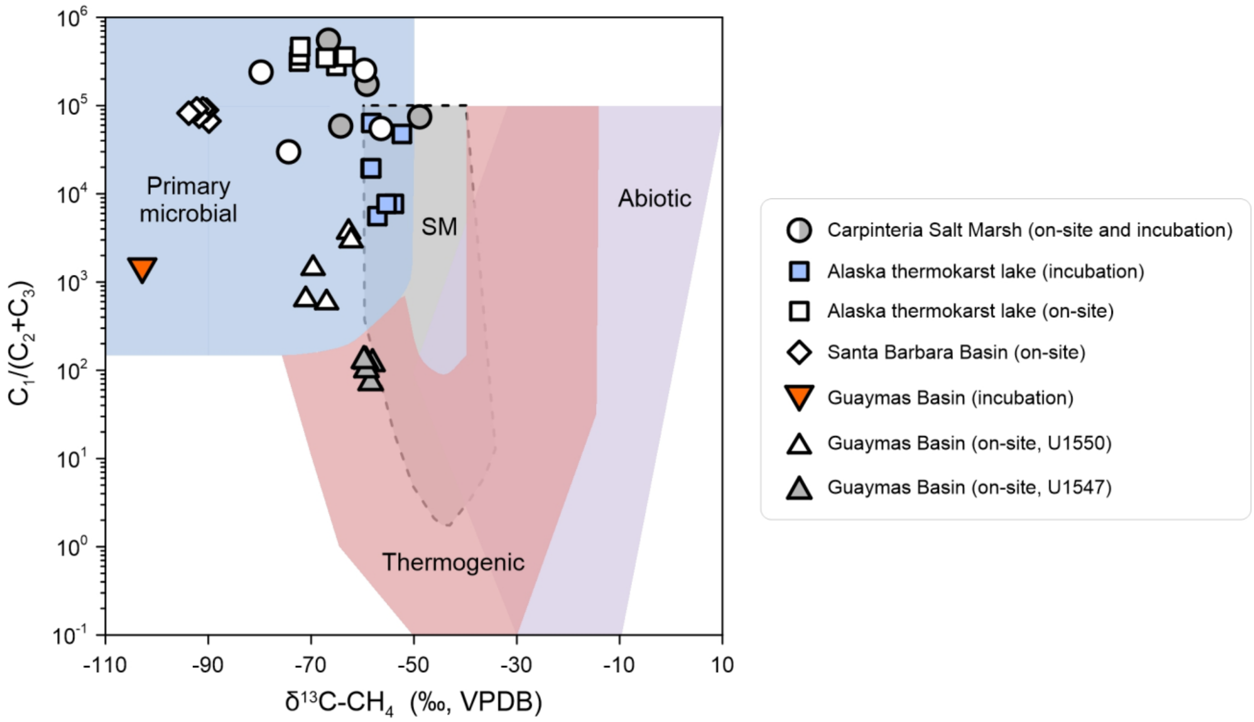

Fig. S13.

$\delta^{13}C_{CH4}$ plotted against $C_1/(C_2 + C_3)$. Genetic fields follow (*7*, *122*). Secondary microbial (SM) field is shown in grey with dashed line. The $C_1/(C_2 + C_3)$ ratios of on-site gas from the Guaymas Basin were adopted from (*85*).

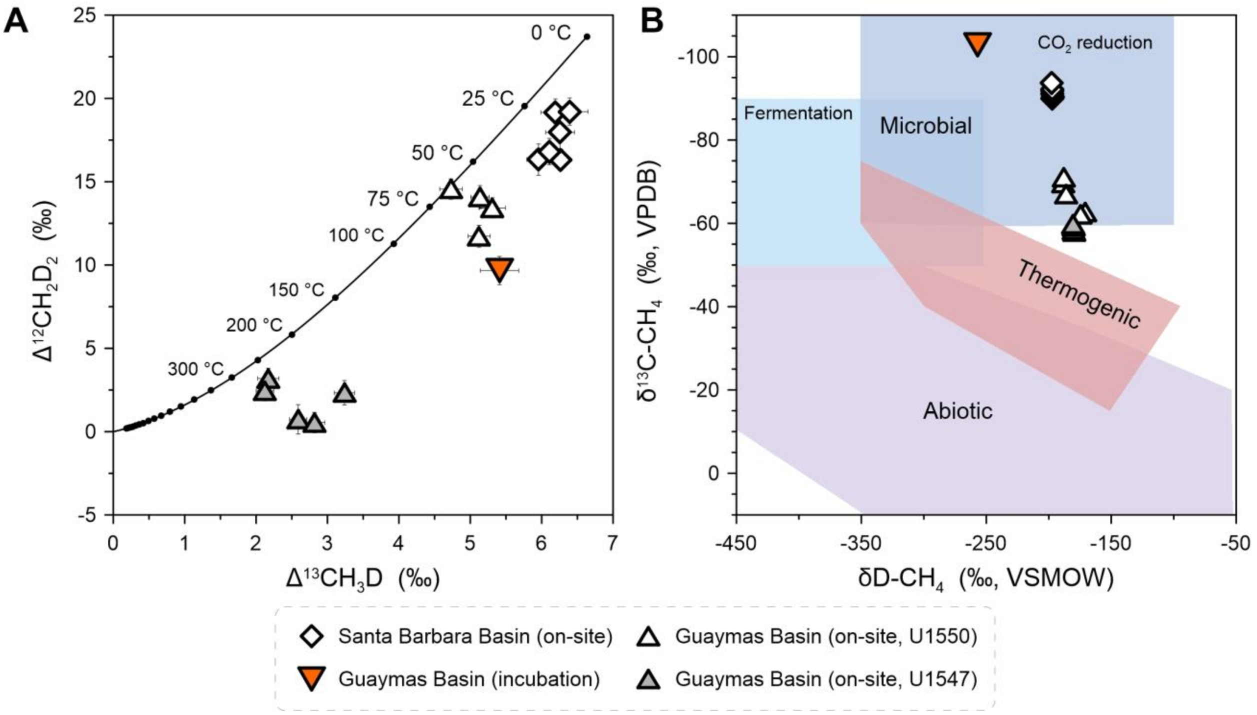

Fig. S14.

Isotopologue compositions of methane samples from deep-sea settings. The same data are presented in Fig. 1, alongside non-microbial methane data obtained from Site U1547 in the Guaymas Basin for comparison.

Supplementary Tables S1–S4

Table S1.

Details describing the sampling sites. Data of Alaska Lakes from (43, 91). Data of Guaymas Basin Sites U1550 and U1547 from (85).

Site	Latitude/longitude	Water depth (m)	Temperature (ºC)
Carpinteria Salt Marsh Site 1	34°24'12.8"N/119°32'07.0"W	0.1–0.2*	19†
Carpinteria Salt Marsh Site 2	34°24'10.2"N/119°32'01.6"W	0.1–0.2*	19†
Alaska Big Trail Lake	64°55'09.5"N/147°49'20.1"W	0.6–2.5	4
Alaska Goldstream Lake	64°54'57.6"N/147°50'52.8"W	1.6–3.3	4
Santa Barbara Basin Site NDRO	34°15'43.8"N/120°01'50.0"W	580	6
Guaymas Basin Seep Site	27°24'18.4"N/111°19'17.2"W	1813	20‡
Guaymas Basin Site U1550	27°15'09.6"N/111°30'25.0"W	2001	16–31
Guaymas Basin Site U1547	27°30'24.8"N/111°40'44.1"W	1732	55–100

*Water depth during low tide.

†Sediment temperature during sampling season.

‡Incubation temperature.

Table S2.

Rates of anaerobic oxidation of methane (AOM) in sediment slurry incubations determined by ^{14}C-radiotracer techniques. All these slurries initially had an argon headspace and accumulated methane over time. Mb and H_2S stand for molybdate and aqueous sulfide, respectively.

Site	Amendment	AOM rate (nmol cm^{-3} d^{-1})
Carpinteria Salt Marsh Site 1	with Mb & H_2S	0
Carpinteria Salt Marsh Site 2	with Mb & H_2S	0
Guaymas Basin Seep Site	with Mb & H_2S	0
Guaymas Basin Seep Site	no amendment	19

Table S3.

Gibbs free energies (ΔG) of methanogenic catabolic pathways at the study sites. Possible ranges of ΔG were determined for the sampling depth of methane isotopologue analysis.

Site	Depth	Substrate	ΔG (kJ mol^{-1} C)
Carpinteria Salt Marsh Sites 1&2	0–5 cm	Methanol	-64.3 to -58.1
Carpinteria Salt Marsh Sites 1&2	0–5 cm	Methylamine	-69.1 to -59.4
Carpinteria Salt Marsh Sites 1&2*	incubation	H_2/CO_2	-113.5
Santa Barbara Basin Site NDRO	174–214 cm	H_2/CO_2	-22.6 to -14.7
Guaymas Basin Site U1550	94–204 m	H_2/CO_2	-36.2 to -25.1
Guaymas Basin Site U1547	82–166 m	H_2/CO_2	-6.1 to 36.5

*Incubation experiments with H_2.

Table S4.

Comparison between energy-rich and energy-limited sites. All data in this table were obtained from published studies and are referenced in the text.

Study site	Volumetric organic carbon oxidation rate (nmol cm^{-3} d^{-1})*	Areal SRR rate (mmol m^{-2} d^{-1})	Areal organic carbon oxidation rate (mmol m^{-2} d^{-1})**
Carpinteria salt marsh	230–3230	11.0–42.2	>22.0–84.4
Santa Barbara Basin	<0.6	1.7–4.1	3.4–8.2
Guaymas Basin	<2	1.4	3.8

*At the depth where on-site methane samples were collected.

**Representing hypothetical minimum.

*,**Assuming a stoichiometric ratio of 2 during organic carbon mineralization to sulfate reduction and methanogenesis (*123*).

Data S1. (separate file)

Research data.

REFERENCES AND NOTES

1. M. Saunois, A. R. Stavert, B. Poulter, P. Bousquet, J. G. Canadell, R. B. Jackson, P. A. Raymond, E. J. Dlugokencky, S. Houweling, P. K. Patra, P. Ciais, V. K. Arora, D. Bastviken, P. Bergamaschi, D. R. Blake, G. Brailsford, L. Bruhwiler, K. M. Carlson, M. Carrol, S. Castaldi, N. Chandra, C. Crevoisier, P. M. Crill, K. Covey, C. L. Curry, G. Etiope, C. Frankenberg, N. Gedney, M. I. Hegglin, L. Höglund-Isaksson, G. Hugelius, M. Ishizawa, A. Ito, G. Janssens-Maenhout, K. M. Jensen, F. Joos, T. Kleinen, P. B. Krummel, R. L. Langenfelds, G. G. Laruelle, L. Liu, T. Machida, S. Maksyutov, K. C. McDonald, J. McNorton, P. A. Miller, J. R. Melton, I. Morino, J. Müller, F. Murguia-Flores, V. Naik, Y. Niwa, S. Noce, S. O'Doherty, R. J. Parker, C. Peng, S. Peng, G. P. Peters, C. Prigent, R. Prinn, M. Ramonet, P. Regnier, W. J. Riley, J. A. Rosentreter, A. Segers, I. J. Simpson, H. Shi, S. J. Smith, L. P. Steele, B. F. Thornton, H. Tian, Y. Tohjima, F. N. Tubiello, A. Tsuruta, N. Viovy, A. Voulgarakis, T. S. Weber, M. van Weele, G. R. van der Werf, R. F. Weiss, D. Worthy, D. Wunch, Y. Yin, Y. Yoshida, W. Zhang, Z. Zhang, Y. Zhao, B. Zheng, Q. Zhu, Q. Zhu, Q. Zhuang, The global methane budget 2000–2017. *Earth Syst. Sci. Data* **12**, 1561–1623 (2020).

2. E. J. Dlugokencky, E. G. Nisbet, R. Fisher, D. Lowry, Global atmospheric methane: Budget, changes and dangers. *Philos. Trans. A Math. Phys. Eng. Sci.* **369**, 2058–2072 (2011).

3. R. Conrad, The global methane cycle: Recent advances in understanding the microbial processes involved. *Environ. Microbiol. Rep.* **1**, 285–292 (2009).

4. S. Schwietzke, O. A. Sherwood, L. M. P. Bruhwiler, J. B. Miller, G. Etiope, E. J. Dlugokencky, S. E. Michel, V. A. Arling, B. H. Vaughn, J. W. C. White, P. P. Tans, Upward revision of global fossil fuel methane emissions based on isotope database. *Nature* **538**, 88–91 (2016).

5. M. J. Whiticar, Carbon and hydrogen isotope systematics of bacterial formation and oxidation of methane. *Chem. Geol.* **161**, 291–314 (1999).

6. E. G. Nisbet, E. J. Dlugokencky, M. R. Manning, D. Lowry, R. E. Fisher, J. L. France, S. E. Michel, J. B. Miller, J. W. C. White, B. Vaughn, P. Bousquet, J. A. Pyle, N. J. Warwick, M. Cain, R. Brownlow, G. Zazzeri, M. Lanoisellé, A. C. Manning, E. Gloor, D. E. J. Worthy,

E.-G. Brunke, C. Labuschagne, E. W. Wolff, A. L. Ganesan, Rising atmospheric methane: 2007–2014 growth and isotopic shift. *Global Biogeochem. Cycles* **30**, 1356–1370 (2016).

7. A. V. Milkov, G. Etiope, Revised genetic diagrams for natural gases based on a global dataset of >20,000 samples. *Org. Geochem.* **125**, 109–120 (2018).

8. D. A. Stolper, M. Lawson, C. L. Davis, A. A. Ferreira, E. V. S. Neto, G. S. Ellis, M. D. Lewan, A. M. Martini, Y. Tang, M. Schoell, A. L. Sessions, J. M. Eiler, Formation temperatures of thermogenic and biogenic methane. *Science* **344**, 1500–1503 (2014).

9. D. T. Wang, D. S. Gruen, B. S. Lollar, K.-U. Hinrichs, L. C. Stewart, J. F. Holden, A. N. Hristov, J. W. Pohlman, P. L. Morrill, M. Könneke, K. B. Delwiche, E. P. Reeves, C. N. Sutcliffe, D. J. Ritter, J. S. Seewald, J. C. McIntosh, H. F. Hemond, M. D. Kubo, D. Cardace, T. M. Hoehler, S. Ono, Nonequilibrium clumped isotope signals in microbial methane. *Science* **348**, 428–431 (2015).

10. E. D. Young, I. E. Kohl, B. S. Lollar, G. Etiope, D. Rumble, S. Li, M. A. Haghnegahdar, E. A. Schauble, K. A. McCain, D. I. Foustoukos, C. Sutclife, O. Warr, C. J. Ballentine, T. C. Onstott, H. Hosgormez, A. Neubeck, J. M. Marques, I. Pérez-Rodríguez, A. R. Rowe, D. E. LaRowe, C. Magnabosco, L. Y. Yeung, J. L. Ash, L. T. Bryndzia, The relative abundances of resolved $^{12}CH_2D_2$ and $^{13}CH_3D$ and mechanisms controlling isotopic bond ordering in abiotic and biotic methane gases. *Geochim. Cosmochim. Acta* **203**, 235–264 (2017).

11. M. A. Haghnegahdar, J. Sun, N. Hultquist, N. D. Hamovit, N. Kitchen, J. Eiler, S. Ono, S. A. Yarwood, A. J. Kaufman, R. R. Dickerson, A. Bouyon, C. Magen, J. Farquhar, Tracing sources of atmospheric methane using clumped isotopes. *Proc. Natl. Acad. Sci. U.S.A.* **120**, e2305574120 (2023).

12. M. Sivan, T. Röckmann, C. van der Veen, M. E. Popa, Extraction, purification, and clumped isotope analysis of methane (Δ13CDH3 and Δ12CD2H2) from sources and the atmosphere. *Atmos. Meas. Tech.* **17**, 2687–2705 (2024).

13. H. Xie, G. Dong, M. Formolo, M. Lawson, J. Liu, F. Cong, X. Mangenot, Y. Shuai, C. Ponton, J. Eiler, The evolution of intra- and inter-molecular isotope equilibria in natural gases with thermal maturation. *Geochim. Cosmochim. Acta* **307**, 22–41 (2021).

14. P. M. J. Douglas, R. Gonzalez Moguel, K. M. Walter Anthony, M. Wik, P. M. Crill, K. S. Dawson, D. A. Smith, E. Yanay, M. K. Lloyd, D. A. Stolper, J. M. Eiler, A. L. Sessions, Clumped isotopes link older carbon substrates with slower rates of methanogenesis in northern lakes. *Geophys. Res. Lett.* **47**, e2019GL086756 (2020).

15. J. L. Ash, M. Egger, T. Treude, I. Kohl, B. Cragg, R. J. Parkes, C. P. Slomp, B. Sherwood Lollar, E. D. Young, Exchange catalysis during anaerobic methanotrophy revealed by 12CH2D2 and 13CH3D in methane. *Geochem. Perspect. Lett.* **10**, 26–30 (2019).

16. E. Lalk, T. Pape, D. S. Gruen, N. Kaul, J. S. Karolewski, G. Bohrmann, S. Ono, Clumped methane isotopologue-based temperature estimates for sources of methane in marine gas hydrates and associated vent gases. *Geochim. Cosmochim. Acta* **327**, 276–297 (2022).

17. E. D. Young, "A two-dimensional perspective on CH4 isotope clumping: Distinguishing process from source," in *Deep Carbon: Past to Present*, B. N. Orcutt, I. Daniel, R. Dasgupta, Eds. (Cambridge Univ. Press, 2019), pp. 388–414.

18. J. Liu, T. Treude, O. R. Abbasov, E. E. Baloglanov, A. A. Aliyev, C. M. Harris, W. D. Leavitt, E. D. Young, Clumped isotope evidence for microbial alteration of thermogenic methane in terrestrial mud volcanoes. *Geology* **52**, 22–26 (2024).

19. X. Wang, B. Chen, H. Nai, C.-Q. Liu, G. Dong, N. Zhang, S.-L. Li, J. Gropp, J. McIntosh, R. M. Ellam, J. M. Eiler, S. Xu, Clumped isotopes constrain thermogenic and secondary microbial methane origins in coal bed methane. *Earth Planet. Sci. Lett.* **647**, 119023 (2024).

20. A. C. Turner, R. Korol, D. L. Eldridge, M. Bill, M. E. Conrad, T. F. Miller, D. A. Stolper, Experimental and theoretical determinations of hydrogen isotopic equilibrium in the system CH_4–H_2–H_2O from 3 to 200°C. *Geochim. Cosmochim. Acta* **314**, 223–269 (2021).

21. D. L. Valentine, A. Chidthaisong, A. Rice, W. S. Reeburgh, S. C. Tyler, Carbon and hydrogen isotope fractionation by moderately thermophilic methanogens. *Geochim. Cosmochim. Acta* **68**, 1571–1590 (2004).

22. D. A. Stolper, A. M. Martini, M. Clog, P. M. Douglas, S. S. Shusta, D. L. Valentine, A. L. Sessions, J. M. Eiler, Distinguishing and understanding thermogenic and biogenic sources of methane using multiply substituted isotopologues. *Geochim. Cosmochim. Acta* **161**, 219–247 (2015).

23. Y. Shuai, H. Xie, S. Zhang, Y. Zhang, J. M. Eiler, Recognizing the pathways of microbial methanogenesis through methane isotopologues in the subsurface biosphere. *Earth Planet. Sci. Lett.* **566**, 116960 (2021).

24. D. Mayumi, H. Tamaki, S. Kato, K. Igarashi, E. Lalk, Y. Nishikawa, H. Minagawa, T. Sato, S. Ono, Y. Kamagata, S. Sakata, Hydrogenotrophic methanogens overwrite isotope signals of subsurface methane. *Science* **386**, 1372–1376 (2024).

25. T. Giunta, E. D. Young, O. Warr, I. Kohl, J. L. Ash, A. Martini, S. O. C. Mundle, D. Rumble, I. Pérez-Rodríguez, M. Wasley, D. E. LaRowe, A. Gilbert, B. Sherwood Lollar, Methane sources and sinks in continental sedimentary systems: New insights from paired clumped isotopologues $^{13}CH_3D$ and $^{12}CH_2D_2$. *Geochim. Cosmochim. Acta* **245**, 327–351 (2019).

26. M. Y. Yoshinaga, T. Holler, T. Goldhammer, G. Wegener, J. W. Pohlman, B. Brunner, M. M. M. Kuypers, K.-U. Hinrichs, M. Elvert, Carbon isotope equilibration during sulphate-limited anaerobic oxidation of methane. *Nat. Geosci.* **7**, 190–194 (2014).

27. G. Wegener, J. Gropp, H. Taubner, I. Halevy, M. Elvert, Sulfate-dependent reversibility of intracellular reactions explains the opposing isotope effects in the anaerobic oxidation of methane. *Sci. Adv.* **7**, eabe4939 (2021).

28. T. Holler, G. Wegener, H. Niemann, C. Deusner, T. G. Ferdelman, A. Boetius, B. Brunner, F. Widdel, Carbon and sulfur back flux during anaerobic microbial oxidation of methane and coupled sulfate reduction. *Proc. Natl. Acad. Sci. U.S.A.* **108**, E1484–E1490 (2011).

29. T. M. Hoehler, B. B. Jørgensen, Microbial life under extreme energy limitation. *Nat. Rev. Microbiol.* **11**, 83–94 (2013).

30. J. J. Middelburg, A simple rate model for organic matter decomposition in marine sediments. *Geochim. Cosmochim. Acta* **53**, 1577–1581 (1989).

31. B. B. Jørgensen, Sulfur biogeochemical cycle of marine sediments. *Geochem. Perspect.* **10**, 145–146 (2021).

32. J. Liu, "The biogeochemistry of methane cycling and its clumped isotope effects," thesis, University of California, Los Angeles (2024).

33. D. S. Gruen, D. T. Wang, M. Könneke, B. D. Topçuoğlu, L. C. Stewart, T. Goldhammer, J. F. Holden, K.-U. Hinrichs, S. Ono, Experimental investigation on the controls of clumped isotopologue and hydrogen isotope ratios in microbial methane. *Geochim. Cosmochim. Acta* **237**, 339–356 (2018).

34. L. Taenzer, J. Labidi, A. L. Masterson, X. Feng, D. Rumble, E. D. Young, W. D. Leavitt, Low $\Delta^{12}CH_2D_2$ values in microbialgenic methane result from combinatorial isotope effects. *Geochim. Cosmochim. Acta* **285**, 225–236 (2020).

35. J. Gropp, Q. Jin, I. Halevy, Controls on the isotopic composition of microbial methane. *Sci. Adv.* **8**, eabm5713 (2022).

36. S. Ono, J. H. Rhim, E. C. Ryberg, Rate limits and isotopologue fractionations for microbial methanogenesis examined with combined pathway protein cost and isotopologue flow network models. *Geochim. Cosmochim. Acta* **325**, 296–315 (2022).

37. X. Cao, H. Bao, Y. Peng, A kinetic model for isotopologue signatures of methane generated by biotic and abiotic CO2 methanation. *Geochim. Cosmochim. Acta* **249**, 59–75 (2019).

38. S. J. E. Krause, T. Treude, Deciphering cryptic methane cycling: Coupling of methylotrophic methanogenesis and anaerobic oxidation of methane in hypersaline coastal wetland sediment. *Geochim. Cosmochim. Acta* **302**, 160–174 (2021).

39. R. S. Oremland, S. Polcin, Methanogenesis and sulfate reduction: Competitive and noncompetitive substrates in estuarine sediments. *Appl. Environ. Microbiol.* **44**, 1270–1276 (1982).

40. M. K. Lloyd, D. L. Eldridge, D. A. Stolper, Clumped $^{13}CH_2D$ and $^{12}CHD_2$ compositions of methyl groups from wood and synthetic monomers: Methods, experimental and theoretical calibrations, and initial results. *Geochim. Cosmochim. Acta* **297**, 233–275 (2021).

41. R. Conrad, Importance of hydrogenotrophic, aceticlastic and methylotrophic methanogenesis for methane production in terrestrial, aquatic and other anoxic environments: A mini review. *Pedosphere* **30**, 25–39 (2020).

42. S. Schulz, R. Conrad, Influence of temperature on pathways to methane production in the permanently cold profundal sediment of Lake Constance. *FEMS Microbiol. Ecol.* **20**, 1–14 (1996).

43. A. Pellerin, N. Lotem, K. Walter Anthony, E. Eliani Russak, N. Hasson, H. Røy, J. P. Chanton, O. Sivan, Methane production controls in a young thermokarst lake formed by abrupt permafrost thaw. *Glob. Chang. Biol.* **28**, 3206–3221 (2022).

44. O. C. Chan, P. Claus, P. Casper, A. Ulrich, T. Lueders, R. Conrad, Vertical distribution of structure and function of the methanogenic archaeal community in Lake Dagow sediment. *Environ. Microbiol.* **7**, 1139–1149 (2005).

45. E. R. C. Hornibrook, F. J. Longstaffe, W. S. Fyfe, Spatial distribution of microbial methane production pathways in temperate zone wetland soils: Stable carbon and hydrogen isotope evidence. *Geochim. Cosmochim. Acta* **61**, 745–753 (1997).

46. K. Walter Anthony, T. Schneider von Deimling, I. Nitze, S. Frolking, A. Emond, R. Daanen, P. Anthony, P. Lindgren, B. Jones, G. Grosse, 21st-century modeled permafrost carbon emissions accelerated by abrupt thaw beneath lakes. *Nat. Commun.* **9**, 3262 (2018).

47. F. Beulig, H. Røy, C. Glombitza, B. B. Jørgensen, Control on rate and pathway of anaerobic organic carbon degradation in the seabed. *Proc. Natl. Acad. Sci. U.S.A.* **115**, 367–372 (2018).

48. R. S. Oremland, B. F. Taylor, Sulfate reduction and methanogenesis in marine sediments. *Geochim. Cosmochim. Acta* **42**, 209–214 (1978).

49. A. Pellerin, G. Antler, H. Røy, A. Findlay, F. Beulig, C. Scholze, A. V. Turchyn, B. B. Jørgensen, The sulfur cycle below the sulfate-methane transition of marine sediments. *Geochim. Cosmochim. Acta* **239**, 74–89 (2018).

50. D. E. Canfield, R. Raiswell, S. H. Bottrell, The reactivity of sedimentary iron minerals toward sulfide. *Am. J. Sci.* **292**, 659–683 (1992).

51. F. Beulig, F. Schubert, R. R. Adhikari, C. Glombitza, V. B. Heuer, K. U. Hinrichs, K. L. Homola, F. Inagaki, B. B. Jørgensen, J. Kallmeyer, S. J. E. Krause, Y. Morono, J. Sauvage, A. J. Spivack, T. Treude, Rapid metabolism fosters microbial survival in the deep, hot subseafloor biosphere. *Nat. Commun.* **13**, 312 (2022).

52. J. H. Rhim, S. Ono, Combined carbon, hydrogen, and clumped isotope fractionations reveal differential reversibility of hydrogenotrophic methanogenesis in laboratory cultures. *Geochim. Cosmochim. Acta* **335**, 383–399 (2022).

53. A. Boetius, K. Ravenschlag, C. J. Schubert, D. Rickert, F. Widdel, A. Gieseke, R. Amann, B. B. Jørgensen, U. Witte, O. Pfannkuche, A marine microbial consortium apparently mediating anaerobic oxidation of methane. *Nature* **407**, 623–626 (2000).

54. T. Giunta, E. D. Young, J. Labidi, P. Sansjofre, D. Jézéquel, J.-P. Donval, C. Brandily, L. Ruffine, Extreme methane clumped isotopologue bio-signatures of aerobic and anaerobic methanotrophy: Insights from the Lake Pavin and the Black Sea sediments. *Geochim. Cosmochim. Acta* **338**, 34–53 (2022).

55. J. Liu, R. L. Harris, J. L. Ash, J. G. Ferry, S. J. E. Krause, J. Labidi, D. Prakash, B. Sherwood Lollar, T. Treude, O. Warr, E. D. Young, Reversibility controls on extreme methane clumped isotope signatures from anaerobic oxidation of methane. *Geochim. Cosmochim. Acta* **348**, 165–186 (2023).

56. G. R. Coon, P. D. Duesing, R. Paul, J. A. Baily, K. G. Lloyd, Biological methane production and accumulation under sulfate-rich conditions at Cape Lookout Bight, NC. *Front. Microbiol.* **14**, 1268361 (2023).

57. S. Arndt, B. B. Jørgensen, D. E. LaRowe, J. J. Middelburg, R. D. Pancost, P. Regnier, Quantifying the degradation of organic matter in marine sediments: A review and synthesis. *Earth Sci. Rev.* **123**, 53–86 (2013).

58. M. Pekař, Affinity and reaction rates: Reconsideration of theoretical background and modelling results. *Zeitschrift für Naturforschung A* **64**, 289–299 (2009).

59. D. A. Beard, H. Qian, Relationship between thermodynamic driving force and one-way fluxes in reversible processes. *PLOS ONE* **2**, e144 (2007).

60. A. W. Dale, P. Regnier, N. J. Knab, B. B. Jørgensen, P. Van Cappellen, Anaerobic oxidation of methane (AOM) in marine sediments from the Skagerrak (Denmark): II. Reaction-transport modeling. *Geochim. Cosmochim. Acta* **72**, 2880–2894 (2008).

61. Y.-S. Lin, V. B. Heuer, T. Goldhammer, M. Y. Kellermann, M. Zabel, K.-U. Hinrichs, Towards constraining H2 concentration in subseafloor sediment: A proposal for combined analysis by two distinct approaches. *Geochim. Cosmochim. Acta* **77**, 186–201 (2012).

62. D. Meier, S. van Grinsven, A. Michel, P. Eickenbusch, C. Glombitza, X. Han, A. Fiskal, S. Bernasconi, C. J. Schubert, M. A. Lever, Hydrogen–independent CO2 reduction dominates methanogenesis in five temperate lakes that differ in trophic states. *ISME Communications* **4**, (2024).

63. E. D. Young, J. Labidi, I. E. Kohl, "Advances in measuring multiply-substituted isotopologues of gas molecules with geochemical applications," in *Treatise on Geochemistry (Third edition)*, A. Anbar, D. Weis, Eds. (Elsevier, 2025), pp. 645–670.

64. R. S. Oremland, D. G. Capone, "Use of "specific" inhibitors in biogeochemistry and microbial ecology," in *Advances in Microbial Ecology*, K. C. Marshall, Ed. (Springer US, 1988), pp. 285–383.

65. E. J. Beal, C. H. House, V. J. Orphan, Manganese-and iron-dependent marine methane oxidation. *Science* **325**, 184–187 (2009).

66. P. N. Froelich, G. P. Klinkhammer, M. L. Bender, N. A. Luedtke, G. R. Heath, D. Cullen, P. Dauphin, D. Hammond, B. Hartman, V. Maynard, Early oxidation of organic matter in pelagic sediments of the eastern equatorial Atlantic: Suboxic diagenesis. *Geochim. Cosmochim. Acta* **43**, 1075–1090 (1979).

67. K. Laufer, A. B. Michaud, H. Røy, B. B. Jørgensen, Reactivity of iron minerals in the seabed toward microbial reduction—A comparison of different extraction techniques. *Geomicrobiol. J.* **37**, 170–189 (2020).

68. J. Liu, E. Klonicki-Ference, S. J. E. Krause, T. Treude, Iron oxides fuel anaerobic oxidation of methane in the presence of sulfate in hypersaline coastal wetland sediment. *Environ. Sci. Technol.* **59**, 513–522 (2025).

69. I. W. Koster, A. Rinzema, A. L. de Vegt, G. Lettinga, Sulfide inhibition of the methanogenic activity of granular sludge at various pH-levels. *Water Res.* **20**, 1561–1567 (1986).

70. F. Widdel, F. Bak, "Gram-negative mesophilic sulfate-reducing bacteria," in *The Prokaryotes: A Handbook on the Biology of Bacteria: Ecophysiology, Isolation, Identification, Applications*, A. Balows, H. G. Trüper, M. Dworkin, W. Harder, K.-H. Schleifer, Eds. (Springer New York, 1992), pp. 3352–3378.

71. A. Vigneron, P. Cruaud, P. Pignet, J.-C. Caprais, M.-A. Cambon-Bonavita, A. Godfroy, L. Toffin, Archaeal and anaerobic methane oxidizer communities in the Sonora Margin cold seeps, Guaymas Basin (Gulf of California). *ISME J.* **7**, 1595–1608 (2013).

72. N. Lotem, A. Pellerin, K. W. Anthony, A. Gafni, V. Boyko, O. Sivan, Anaerobic oxidation of methane does not attenuate methane emissions from thermokarst lakes. *Limnol. Oceanogr.* **68**, 1316–1330 (2023).

73. J. D. Cline, Spectrophotometric determination of hydrogen sulfide in natural waters. *Limnol. Oceanogr.* **14**, 454–458 (1969).

74. L. L. Stookey, Ferrozine—A new spectrophotometric reagent for iron. *Anal. Chem.* **42**, 779–781 (1970).

75. T. M. Hoehler, M. J. Alperin, D. B. Albert, C. S. Martens, Thermodynamic control on hydrogen concentrations in anoxic sediments. *Geochim. Cosmochim. Acta* **62**, 1745–1756 (1998).

76. T. E. Crozier, S. Yamamoto, Solubility of hydrogen in water, sea water, and sodium chloride solutions. *J. Chem. Eng. Data* **19**, 242–244 (1974).

77. B. G. Kopec, X. Feng, E. S. Posmentier, L. J. Sonder, Seasonal deuterium excess variations of precipitation at Summit, Greenland, and their climatological significance. *J. Geophys. Res. Atmos.* **124**, 72–91 (2019).

78. X. Cui, A. Mucci, T. S. Bianchi, D. He, D. Vaughn, E. K. Williams, C. Wang, C. Smeaton, K. Koziorowska-Makuch, J. C. Faust, A. F. Plante, B. E. Rosenheim, Global fjords as transitory reservoirs of labile organic carbon modulated by organo-mineral interactions. *Sci. Adv.* **8**, eadd0610 (2022).

79. J. D. Hemingway, D. H. Rothman, S. Z. Rosengard, V. V. Galy, Technical note: An inverse method to relate organic carbon reactivity to isotope composition from serial oxidation. *Biogeosciences* **14**, 5099–5114 (2017).

80. S. Yamamoto, J. B. Alcauskas, T. E. Crozier, Solubility of methane in distilled water and seawater. *J. Chem. Eng. Data* **21**, 78–80 (1976).

81. S. Krause, R. Wipfler, J. Liu, D. J. Yousavich, D. Robinson, D. W. Hoyt, V. J. Orphan, T. Treude, Spatial evidence of cryptic methane cycling and methylotrophic metabolisms along a land-ocean transect in a California coastal wetland. bioRxiv 603764 [Preprint] (2024). https://doi.org/10.1101/2024.07.16.603764.

82. P. Berg, N. Risgaard-Petersen, S. Rysgaard, Interpretation of measured concentration profiles in sediment pore water. *Limnol. Oceanogr.* **43**, 1500–1510 (1998).

83. E. R. Sholkovitz, J. M. Gieskes, A physical-chemical study of the flushing of the Santa Barbara Basin. *Limnol. Oceanogr.* **16**, 479–489 (1971).

84. C. Li, A. L. Sessions, F. S. Kinnaman, D. L. Valentine, Hydrogen-isotopic variability in lipids from Santa Barbara Basin sediments. *Geochim. Cosmochim. Acta* **73**, 4803–4823 (2009).

85. A. Teske, D. Lizarralde, T. W. Höfig, "The Expedition 385 Scientists," in *Guaymas Basin Tectonics and Biosphere. Proceedings of the International Ocean Discovery Program* (International Ocean Discovery Program, 2021).

86. J. W. Johnson, E. H. Oelkers, H. C. Helgeson, SUPCRT92: A software package for calculating the standard molal thermodynamic properties of minerals, gases, aqueous species, and reactions from 1 to 5000 bar and 0 to 1000°C. *Comput. Geosci.* **18**, 899–947 (1992).

87. E. R. Lewis, D. W. R. Wallace, *Program Developed for CO2 System Calculations* (Carbon Dioxide Information Analysis Center, Oak Ridge National Laboratory, US Department of Energy, Oak Ridge, TN, USA, 1998).

88. J. P. Amend, D. E. LaRowe, Minireview: Demystifying microbial reaction energetics. *Environ. Microbiol.* **21**, 3539–3547 (2019).

89. D. P. Bojanova, V. Y. De Anda, M. A. Haghnegahdar, A. P. Teske, J. L. Ash, E. D. Young, B. J. Baker, D. E. LaRowe, J. P. Amend, Well-hidden methanogenesis in deep, organic-rich sediments of Guaymas Basin. *ISME J.* **17**, 1828–1838 (2023).

90. H. M. Page, R. L. Petty, D. E. Meade, Influence of watershed runoff on nutrient dynamics in a Southern California salt marsh. *Estuar. Coast. Shelf Sci.* **41**, 163–180 (1995).

91. K. M. Walter Anthony, P. Lindgren, P. Hanke, M. Engram, P. Anthony, R. P. Daanen, A. Bondurant, A. K. Liljedahl, J. Lenz, G. Grosse, B. M. Jones, L. Brosius, S. R. James, B. J. Minsley, N. J. Pastick, J. Munk, J. P. Chanton, C. E. Miller, F. J. Meyer, Decadal-scale hotspot methane ebullition within lakes following abrupt permafrost thaw. *Environ. Res. Lett.* **16**, 035010 (2021).

92. T. M. Hill, J. P. Kennett, H. J. Spero, High-resolution records of methane hydrate dissociation: ODP Site 893, Santa Barbara Basin. *Earth Planet. Sci. Lett.* **223**, 127–140 (2004).

93. S. J. E. Krause, J. Liu, D. J. Yousavich, D. Robinson, D. W. Hoyt, Q. Qin, F. Wenzhöfer, F. Janssen, D. L. Valentine, T. Treude, Evidence of cryptic methane cycling and non-methanogenic methylamine consumption in the sulfate-reducing zone of sediment in the Santa Barbara Basin, California. *Biogeosciences* **20**, 4377–4390 (2023).

94. S. E. Calvert, Origin of diatom-rich, varved sediments from the Gulf of California. *J. Geol.* **74**, 546–565 (1966).

95. P. Lonsdale, K. Becker, Hydrothermal plumes, hot springs, and conductive heat flow in the Southern Trough of Guaymas Basin. *Earth Planet. Sci. Lett.* **73**, 211–225 (1985).

96. A. Teske, L. J. McKay, A. C. Ravelo, I. Aiello, C. Mortera, F. Núñez-Useche, C. Canet, J. P. Chanton, B. Brunner, C. Hensen, G. A. Ramírez, R. J. Sibert, T. Turner, D. White, C. R. Chambers, A. Buckley, S. B. Joye, S. A. Soule, D. Lizarralde, Characteristics and evolution of sill-driven off-axis hydrothermalism in Guaymas Basin—The Ringvent site. *Sci. Rep.* **9**, 13847 (2019).

97. Q. Jin, C. M. Bethke, Kinetics of electron transfer through the respiratory chain. *Biophys. J.* **83**, 1797–1808 (2002).

98. K. Nauhaus, A. Boetius, M. Krüger, F. Widdel, In vitro demonstration of anaerobic oxidation of methane coupled to sulphate reduction in sediment from a marine gas hydrate area. *Environ. Microbiol.* **4**, 296–305 (2002).

99. G. Wegener, A. Boetius, An experimental study on short-term changes in the anaerobic oxidation of methane in response to varying methane and sulfate fluxes. *Biogeosciences* **6**, 867–876 (2009).

100. F. Beulig, H. Røy, S. E. McGlynn, B. B. Jørgensen, Cryptic CH_4 cycling in the sulfate–methane transition of marine sediments apparently mediated by ANME-1 archaea. *ISME J.* **13**, 250–262 (2019).

101. M. A. Haghnegahdar, E. A. Schauble, E. D. Young, A model for $^{12}CH_2D_2$ and $^{13}CH_3D$ as complementary tracers for the budget of atmospheric CH_4. *Global Biogeochem. Cycles* **31**, 1387–1407 (2017).

102. T. Holler, G. Wegener, K. Knittel, A. Boetius, B. Brunner, M. M. M. Kuypers, F. Widdel, Substantial $^{13}C/^{12}C$ and D/H fractionation during anaerobic oxidation of methane by marine consortia enriched in vitro. *Environ. Microbiol. Rep.* **1**, 370–376 (2009).

103. R. Conrad, Contribution of hydrogen to methane production and control of hydrogen concentrations in methanogenic soils and sediments. *FEMS Microbiol. Ecol.* **28**, 193–202 (1999).

104. J. Rudd, C. D. Taylor, Methane cycling in aquatic environments. *Adv. Aquat. Microbiol.* **2**, 77–150 (1980).

105. G.-C. Zhuang, V. B. Heuer, C. S. Lazar, T. Goldhammer, J. Wendt, V. A. Samarkin, M. Elvert, A. P. Teske, S. B. Joye, K.-U. Hinrichs, Relative importance of methylotrophic methanogenesis in sediments of the Western Mediterranean Sea. *Geochim. Cosmochim. Acta* **224**, 171–186 (2018).

106. J. Maltby, S. Sommer, A. W. Dale, T. Treude, Microbial methanogenesis in the sulfate-reducing zone of surface sediments traversing the Peruvian margin. *Biogeosciences* **13**, 283–299 (2016).

107. K.-Q. Xiao, F. Beulig, H. Røy, B. B. Jørgensen, N. Risgaard-Petersen, Methylotrophic methanogenesis fuels cryptic methane cycling in marine surface sediment. *Limnol. Oceanogr.* **63**, 1519–1527 (2018).

108. M. J. Whiticar, E. Faber, M. Schoell, Biogenic methane formation in marine and freshwater environments: CO_2 reduction vs. acetate fermentation—Isotope evidence. *Geochim. Cosmochim. Acta* **50**, 693–709 (1986).

109. P. M. Crill, C. S. Martens, Methane production from bicarbonate and acetate in an anoxic marine sediment. *Geochim. Cosmochim. Acta* **50**, 2089–2097 (1986).

110. T. Treude, D. L. Valentine, Microbial activity from sediments collected Fall 2019 in the Santa Barbara Basin using ROV Jason during R/V Atlantis cruise AT42-19. Biological and Chemical Oceanography Data Management Office (BCO-DMO). (Version 1) version date 28 December 2021. doi:10.26008/1912/bco-dmo.867221.1. (2022).

111. T. Nagakura, F. Schubert, D. Wagner, J. Kallmeyer, IODP Exp. 385 Shipboard Scientific Party, Biological sulfate reduction in deep subseafloor sediment of Guaymas Basin. *Front. Microbiol.* **13**, 845250 (2022).

112. D. J. Yousavich, D. Robinson, X. Peng, S. J. E. Krause, F. Wenzhöfer, F. Janssen, N. Liu, J. Tarn, F. Kinnaman, D. L. Valentine, T. Treude, Marine anoxia initiates giant sulfur-oxidizing bacterial mat proliferation and associated changes in benthic nitrogen, sulfur, and iron cycling in the Santa Barbara Basin, California Borderland. *Biogeosciences* **21**, 789–809 (2024).

113. A. Weber, B. B. Jørgensen, Bacterial sulfate reduction in hydrothermal sediments of the Guaymas Basin, Gulf of California, Mexico. *Deep-Sea Res. Pt. I* **49**, 827–841 (2002).

114. D. Canfield, *Aquatic Geomicrobiology* (Elsevier Academic Press, 2005), vol. 640.

115. M. Egger, N. Riedinger, J. M. Mogollón, B. B. Jørgensen, Global diffusive fluxes of methane in marine sediments. *Nat. Geosci.* **11**, 421–425 (2018).

116. G. Einsele, J. M. Gieskes, J. Curray, D. M. Moore, E. Aguayo, M.-P. Aubry, D. Fornari, J. Guerrero, M. Kastner, K. Kelts, M. Lyle, Y. Matoba, A. Molina-Cruz, J. Niemitz, J. Rueda, A. Saunders, H. Schrader, B. Simoneit, V. Vacquier, Intrusion of basaltic sills into highly porous sediments, and resulting hydrothermal activity. *Nature* **283**, 441–445 (1980).

117. A. Teske, A. V. Callaghan, D. E. LaRowe, Biosphere frontiers of subsurface life in the sedimented hydrothermal system of Guaymas Basin. *Front. Microbiol.* **5**, 362 (2014).

118. J. E. Hinkle, P. Mara, D. J. Beaudoin, V. P. Edgcomb, A. P. Teske, A PCR-based survey of methane-cycling archaea in methane-soaked subsurface sediments of Guaymas Basin, Gulf of California. *Microorganisms* **11**, 2956 (2023).

119. P. Mara, Y.-L. Zhou, A. Teske, Y. Morono, D. Beaudoin, V. Edgcomb, Microbial gene expression in Guaymas Basin subsurface sediments responds to hydrothermal stress and energy limitation. *ISME J.* **17**, 1907–1919 (2023).

120. G. Dong, H. Xie, M. Formolo, M. Lawson, A. Sessions, J. Eiler, Clumped isotope effects of thermogenic methane formation: Insights from pyrolysis of hydrocarbons. *Geochim. Cosmochim. Acta* **303**, 159–183 (2021).

121. E. Lalk, J. S. Seewald, L. T. Bryndzia, S. Ono, Kilometer-scale $\Delta^{13}CH_3D$ profiles distinguish end-member mixing from methane production in deep marine sediments. *Org. Geochem.* **181**, 104630 (2023).

122. B. Bernard, J. M. Brooks, W. M. Sackett, *Offshore Technology Conference* (Offshore Technology Conference, 1977), pp. 435–438.

123. D. J. Burdige, *Geochemistry of Marine Sediments* (Princeton Univ. Press, 2006).
